# Transcriptional Dissection of Human Limbal Niche Compartments by Massive Parallel Sequencing

**DOI:** 10.1371/journal.pone.0064244

**Published:** 2013-05-22

**Authors:** Chris Bath, Danson Muttuvelu, Jeppe Emmersen, Henrik Vorum, Jesper Hjortdal, Vladimir Zachar

**Affiliations:** 1 Department of Ophthalmology, Aalborg University Hospital, Aalborg, Denmark; 2 Laboratory for Stem Cell Research, Aalborg University, Aalborg, Denmark; 3 Department of Ophthalmology, Aarhus University Hospital, Aarhus, Denmark; Instituto Butantan, Brazil

## Abstract

Corneal epithelium is maintained throughout life by well-orchestrated proliferation of limbal epithelial stem cells (LESCs), followed by migration and maturation centripetally towards the ocular surface. Disturbance of LESCs can potentially lead to a blinding condition, which can be reversed by reconstitution of a functional LESC pool. The current clinical procedures are effective to some degree, however, deeper knowledge of the molecular interplay within the limbal niche is necessary to achieve a fully satisfactory patient outcome. The present study was thus undertaken to carry out a comprehensive transcriptome analysis of four distinct human limbal compartments, including basal limbal crypts (BLCs), superficial limbal crypts (SLCs), cornea, and the supporting stroma, with the aid of laser capture microdissection and deep RNA sequencing. The tissue harvest pipeline was rigorously optimized so that the exposure to cold ischemia would be less than five minutes. The global gene ontology analysis confirmed existence of primitive cells in BLCs, migratory and activated cells in SLCs, and differentiated cells in cornea. Interestingly, many significantly upregulated genes in SLCs mapped to processes involved in regulation of vasculature, such as sFLT1. In contrast, BLCs exhibited many genes mapping to neurogenic processes and processes related to cell development. The primitive nature of BLCs was, furthermore, confirmed by the KEGG pathway analysis, and some potential regulators of LESCs were revealed, such as Lrig1 and SOX9. The analysis also yielded comprehensive lists of uniquely expressed genes in both BLCs and cornea, which may be useful to identify possible biomarkers. In conclusion, the current investigation provides new insight into the relationship between distinct cell populations within the limbal niche, identifies candidates to be verified for novel biological functions, and yields a wealth of information for prospective data mining.

## Introduction

Regenerative medicine is a rapidly developing field promising to cure intractable diseases by adopting both tissue engineering and cell transplantation-based strategies. One of the most successful clinical procedures has been cultured limbal epithelial transplantation (CLET), where the corneal epithelium, damaged due to stem cell dysfunction or depletion, can be repaired using *ex vivo* engineered stem cell grafts. The outcome of surgery is dependent on the graft sheet quality, and thereby the *ex vivo* expansion protocols. The basic culturing approach implemented for CLET [1] was originally developed using epidermal keratinocytes [2], [3]. Significant effort has since been expended to optimize the *ex vivo* propagation of LESCs with the ultimate goal of increasing success rates of the transplantation procedures [4]–[14]. These attempts have been complicated by the lack of stem cell markers and the precise knowledge of molecular regulations involved in the homeostasis of the limbal niche [15].

Corneal epithelium is composed of a multilayered non-keratinized squamous epithelium. Since the early study by Mann (1944) [16], convincing evidence has accumulated to identify corneal limbus as the stem cell reservoir for the continuous replenishment of corneal epithelial cells throughout the lifetime of an individual [17]–[23]. The stem cells are thought to be located in distinct stem cell niches deep in the epithelium specialized in maintaining stem cells, a concept first described by Schofield for the hematopoietic system [24]. In the cornea, these histologically defined structures are termed limbal crypts [23], limbal epithelial crypts [25], or focal stromal projections [23]. Studies have shown, that the limbal epithelial stem cells (LESCs) differentiate as they move away from the basal crypts towards the central cornea, where they migrate superficially, and are ultimately lost to the external environment [18].

Most molecular biological techniques require the desired cell population to be isolated from the surrounding tissue prior to analysis. This has traditionally been performed using either mechanical or enzymatic dissociation with an almost certain change in expression profiles [26]–[28] but also a risk of contamination. A technique, whereby these obstacles can be circumvented, is the use of laser capture microdissection (LCM) [26], which allows separation based on morphological criteria of specific subpopulations from their complex biological environment. LCM is especially suitable for the isolation of corneal epithelial subpopulations due to their unique spatial organization along the differentiation pathway.

A low frequency of stem cells in a given tissue is another problem, frequently associated with stem cell research. The signals from a few stem cells are inevitably lost in the larger pool of mixed cell populations and even after enrichment by LCM, the RNA yield may be too low to allow for global transcriptional profiling using traditional microarrays [29]. The more sensitive massive parallel sequencing thereby provides an optimal tool for investigating complex eukaryotic transcriptomes from sparse stem cell populations. Advantages over traditional microarrays include less bias, lower false positive signals, higher reproducibility, an almost unlimited dynamic range, and a very high sensitivity [29]–[31]. A considerable benefit is also the possibility to identify novel genes and alternative transcripts. Deep RNA sequencing thus lends itself as a method of choice to analyze limited amount of source material such as that from specific limbal compartments.

In the current investigation, we present comprehensive sequence data from the transcriptomes of discrete differentiation stages of corneal epithelium and supporting stroma using a powerful combination of laser capture microdissection and RNA sequencing. In addition to new evidence about molecular networks associated with the limbal niche, the investigation provided an abundance of information for prospective data mining.

## Materials and Methods

### Tissue Acquisition and Processing

The study was approved by The North Denmark Region Committee on Health Research Ethics, and a written informed consent was obtained prior to inclusion. The inclusion criteria stipulated, that the donor should be older than 18 years of age with no history of mental illness. The exclusion criteria specified the absence of both prior radiation therapy against ocular surface, diseases at the ocular surface, and systemic inflammatory diseases. An otherwise normal human eye suffering from malignant melanoma of the retinal pigment epithelium layer was procured, and the corneo-limbal region was isolated and processed immediately after enucleation at the operation theatre to preserve the integrity of material. During all steps along the RNA isolation procedure, only RNase-free reagents were used, the work was restricted to a designated area, surfaces were treated with RNase AWAY (VWR-Bie & Berntsen, Herlev, DK), and a protective clothing was worn. The corneo-limbal tissue was divided by three parallel cuts, each 4 mm apart, into four strips using a custom-made corneal cutting device. The tissue was then stabilized with Tissue-Tek OCT (Sakura Finetek Europe, Alphen aan den Rijn, The Netherlands) inside cryomolds and flash-frozen in a mixture of isopenthane and dry ice. The samples were transported to the laboratory on dry ice, and stored at –140°C until further use.

The tissue blocks were processed into 10 µm serial sections using an HM 505 N cryostat (Microm International, Germany) and mounted on Superfrost Plus glass slides (Thermo Fisher Scientific, Waltham, MA) for phase contrast inspection of niche structures. The selected sections were then transferred onto UV irradiated (3000 µJ/cm^2^) PEN (polyethylene naphtalate)-membrane metal-framed slides (Life Technologies, Naerum, Denmark), and fixed in 70% ethanol at −30°C for 2 min. The staining procedure was completed inside a laminar flow bench using RNase-free pap-jars (Evergreen Scientific, Los Angeles, CA) and reagents pre-cooled to 4°C. In short, the slides were washed with water, counter-stained with 0.01% cresyl violet for 10 sec, and washed with 70% ethanol for 30 sec. Finally, the preparations were dehydrated with 100% ethanol for 2 min and xylene for 5 min. The slides were kept inside the pap-jars at room temperature to avoid condensation of water, which could activate endogenous RNases and interfere with LCM.

### Laser Capture Microdissection and RNA Isolation

The stained tissue sections were sandwiched with Superfrost Plus glass slides to provide a support for dissection and capture in a Veritas microdissection instrument (model 704; Arcturus Bioscience, Mountain View, CA), which was equipped with IR capture and UV cutting lasers. The particular arrangement of the specimen (inverse technique) offered distinctive benefits in minimizing the risk of contamination and enabling both large capture area and constant laser settings. The efficiency of capture thus attained 100%, and the hands-on time to finalize the procedure was reduced greatly. The settings for IR laser pulse were power 100 mW, duration 2.7 msec, and hit frequency 1. The UV laser was set on a constant low power. Four discrete compartments were targeted, the basal limbal crypts (BLCs), the superficial limbal crypts (SLCs), the paracentral/central corneal epithelium, and the adjacent limbal stroma. Each of the four specific areas from a single tissue cryosection was comprehensively sampled and collected on individual CapSure Macro LCM Caps (Life Technologies). A single capture cap was used for up to three cryosections and altogether 15 cryosections were processed. To keep the exposure to less than 1 hour, only three specimens were handled at a time, and upon completion, the thermoplastic film overlaid samples were immediately stripped from the caps to be used for RNA extraction.

The total RNA was isolated using PicoPure RNA isolation kit (Life Technologies) according to the manufactureŕs protocol, including an additional step of DNase I treatment (Sigma-Aldrich, St. Louis, MO), and stored at –140°C until use. The integrity and concentration was determined in the Agilent Bioanalyzer 2100 (Agilent Technologies, Santa Clara, CA) using the Agilent RNA 6000 Pico Kit (Agilent Technologies).

### RNA Amplification and Sequencing

The total RNA was reverse-transcribed and amplified using Ovation RNA-Seq System V2 kit (NuGEN Technologies, San Carlos, CA), and after quantification of dsDNA by the Qubit kit (Life Technologies), the libraries were constructed for paired-end sequencing with the aid of a TruSeq DNA Sample Preparation Kit (Illumina, San Diego, CA). The libraries were quantified by a real-time PCR and further purified employing Agencourt AMPure XP beads (Beckman Coulter, Brea, CA). Finally, to assure optimal loading for cluster generation, the extracted libraries were quantified using a KAPA Library Quant Kit (KAPA Biosystems, Woburn, MA). Sequencing was performed on the Illumina HiSeq2000 platform (Illumina). The sequences were submitted to ENA (European Nucleotide Archive) under accession number E-MTAB-1498.

### Reads Assembly and *in silico* Gene Expression Analysis

The sequencing data were imported as paired-ends into CLC Genomics Workbench 5.5.1 (CLC bio, Aarhus, Denmark) for downstream processing and analysis. Initial trimming of reads was performed using a maximal allowed ambiguity number of 2 within reads, 0.05 limit on quality score, and a removal of the initial ten 5′ base pairs. Assembly of reads was performed against an annotated Homo sapiens hg19 reference genome and followed by a creation of 4 distinct RNA-sequencing experiments, the stroma vs. BLCs, the BLCs vs. SLCs, the SLCs vs. cornea, and the BLCs vs. cornea. Venny (Oliveros, J.C. (2007) VENNY. An interactive tool for comparing lists with Venn Diagrams. http://bioinfogp.cnb.csic.es/tools/venny/index.html) was used to render 4-way interactions among the independent libraries according to selected criteria, and Gene-E (http://www.broadinstitute.org/cancer/software/GENE-E/) was invoked to produce heat maps of RPKM values based on hierarchical clustering.

### Hypergeometric Testing on Annotations and Gene Ontology (GO) Visualization

Hypergeometric tests were performed on annotations using updated databases from The Gene Ontology for GO categories from Biological Process and Kyoto Encyclopedia of Genes and Genomes for pathways in ClueGO [32]. Only genes with FDR corrected p (q) ≤0.05 and a fold-regulation >2 in a pairwise compartment comparison were included in the hypergeometric testing. The ClueGO output was restricted to GO categories with q ≤0.05, and the GO term fusion was used to avoid redundancy prior to visualization of networks in the Cytoscape environment [33]. Grouping of terms was based on kappa statistics, and the leading term was highlighted in the networks in italics. For the global analyses of epithelial populations only GO levels from 1 to 10 were allowed, with at least 5 genes per node and a minimal representation of 3% genes for each term. Specific gene lists for global terms were then remapped in a more detailed analysis. No filtering was performed, when significant genes were mapped to the Kyoto Encyclopedia of Genes and Genomes (KEGG) pathways. GraphPad Prism software (GraphPad Software, La Jolla, CA) was used to create the bar charts.

### Statistics

Significance was tested using proportional statistics on total exon reads using method by Kal et al. (1999) [34] as implemented in the CLC software. Stringent criteria were applied, involving q ≤0.05 according to Benjamini and Hochberg [35], fold-regulation >2 for estimation of up- and downregulated transcripts, and also q ≤0.05 for estimation of significant GO categories. The criteria for potential candidate stem cell and differentiation markers in the BLCs and cornea, respectively, stipulated that the detected transcripts must be unique to the tissue, q ≤0.05, and RPKM higher than that of the SLCs used for comparison.

## Results

### Preparation and Evaluation of Compartment-specific Libraries

**Figure 1:**
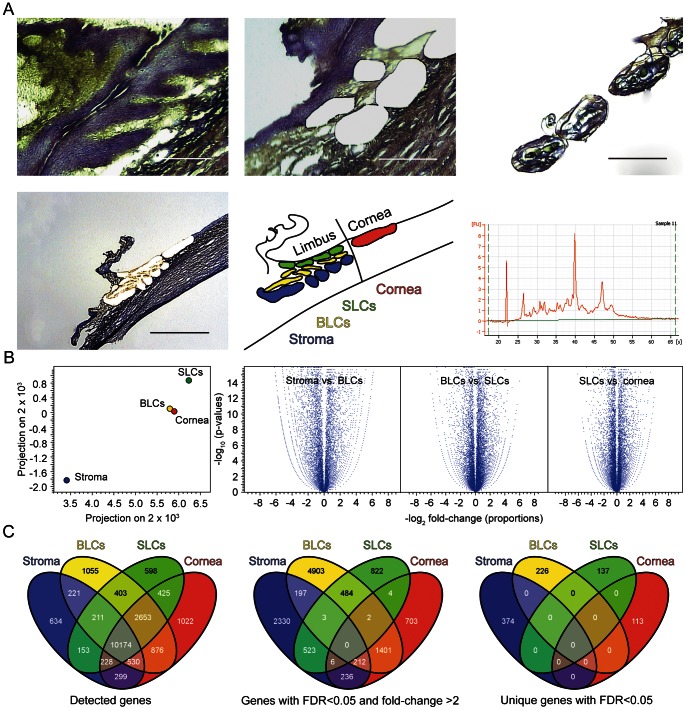
LCM of the corneal limbus and comparison of the compartment-specific libraries. (A) Representative images from the LCM procedure and the electropherogram of the isolated RNA. Scale bars high power magnification, 200 µm; low power magnification, 1 mm. (B) Principal component analysis and volcano plots of the compartment-specific libraries. (C) Distribution of transcriptional expression between limbal compartments using four-way Venn Diagrams.

The spatial arrangement of epithelial subpopulations on the ocular surface allowed for capturing of discrete cellular compartments along the differentiation pathway from BLCs to the superficial cornea. In addition, supporting stroma to this epithelium was successfully retrieved. Representative images of the laser capture procedure along with an electropherogram illustrating integrity of the isolated RNA are shown in figure 1A. The quality of RNA, as expressed by the RIN number, was around six, and the amount of total RNA surpassed 5000 pg in all pooled samples.

The sequencing of the BLC, SLC, cornea, and stroma libraries produced 92,711,354, 85,584,998, 106,506,532, and 86,574,660 paired reads, respectively. Only one third of the reads was found to map to exons (Table S1), which resulted in correspondingly low reads per kilobase per million mapped reads (RPKM) values. This was due to the amplification procedure that employed random SPIA primers. Average read length after trimming the initial 101 bp reads was 86.5 for BLCs, 85.9 for SLCs, 86.2 for cornea, and 86 for stroma. The mapping was performed against 55,075 genes and 162,508 transcripts from Homo sapiens hg19 genome.

### Relatedness and Distribution of Transcriptional Expression between Limbal Compartments

The samples appeared to cluster according to expectations in the principal component analysis, demonstrating relatedness of the epithelial populations away from the stroma (Fig. 1B, left panel). This was further corroborated by hierarchical clustering and scatter plots (data not shown). The volcano plots were symmetrically distributed around 0, and many transcripts were both significantly and differentially expressed between compartments (Fig. 1B, right panel).

Mapping to the Homo sapiens genome (hg19) revealed 16,125 genes expressed in BLCs, 14,847 in SLCs, 16,208 in cornea, and 12,451 in the stroma. The analysis of gene distribution by a four-way Venn diagram plotting indicated that the majority of transcripts were shared, although a limited number of uniquely expressed genes were confirmed as well (Fig. 1C, left panel). After filtering for genes with q ≤0.05 and a fold-change >2, as expected, the corneal subpopulations appeared more disparate (Fig. 1C, middle panel). However, the most accurate information about the prevalence of rare unique transcripts, that potentially may prove to represent novel biomarkers, was obtained after filtering unique genes with q ≤0.05 (Fig. 1C, right panel). The distribution analysis thus confirms, that all compartments have a specific transcriptional signature, and each of them features a set of uniquely expressed genes.

**Figure 2:**
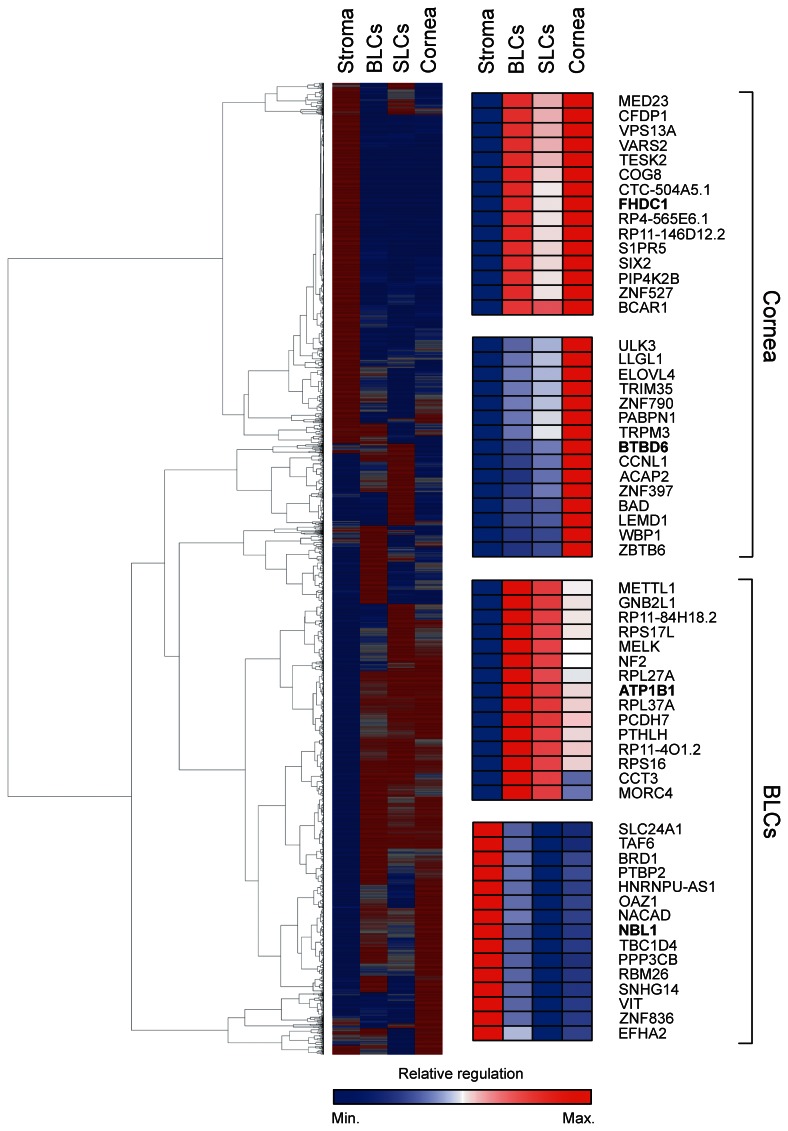
Heatmap of significantly expressed genes in BLCs, SLCs, cornea, and stroma after hierarchical clustering based on RPKM values. Red indicates high, and blue low RPKM. High resolution inserts for FHDC1 and BTBD6, and ATPB1 and NBL1 are presented.

The RPKM is a normalized measure of expression levels in RNA sequencing. Genes with q ≤0.05 and a fold-change >2 were grouped based on RPKM values using the method of hierarchical clustering, and a global heatmap was compiled (Fig. 2). It is clearly evident that distinct subpopulations of corneal cells use specific transcriptional programs that reflect their functional differences.

### GO Analysis of Differentially Expressed Genes in BLCs

**Figure 3:**
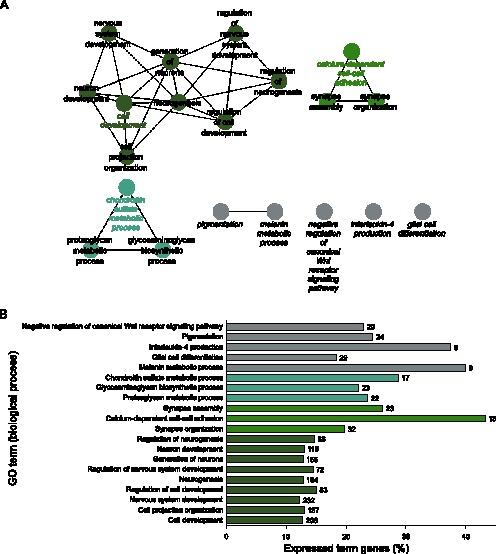
Gene ontology analysis of significantly upregulated genes in BLCs. (A) Network of significantly enriched terms. (B) Bar chart of significantly enriched terms. Numbers next to the bars indicate total number of genes mapping to the specific term.

The association between compartment-specific transcriptional activity and cellular function was examined using Gene Ontology. The analysis was based on statistical significant overrepresentation of categories in the ontology Biological Process. The GO analysis for BLCs revealed the presence of functions indicative of development and differentiation within the corneal context (Fig. 3). This is evident from the fact, that the term *Cell Development* was identified as a group leading term. The genes mapping to this leading term were also remapped in more detail and are available as supplemental information (Fig. S1). In particular, it was possible to identify several processes central to development, such as *Epithelial to Mesenchymal Transition, Mesenchymal Cell Development, Mesenchymal Cell Differentiation,* and *Regulation of Cell Morphogenesis Involved in Differentiation*. From the point of limbal homeostasis it appears important, that the category *Pigmentation* was significantly enriched. The presence of melanocytes in corneal epithelium is restricted to BLCs, where they are presumed to protect the LESCs from UV-induced damage [36]. A more detailed mapping of *Pigmentation* also confirmed the ocular nature of pigmentation as *Eye Pigmentation*.

The prominent attribute of BLCs as a location harboring the LESCs was further substantiated by finding the presumed stem cell markers upregulated and the differentiation markers downregulated when compared with the other compartments. Thus the RPKM values for Homo sapiens tumor protein p63 (transcript variant 4), also known as deltaNp63α, were 16.34 in the BLCs, but 11.94 and 10.42 in the SLCs and cornea, respectively. Similarly, for cytokeratin 19 (KRT19), the values were 47.62 and 62.42 in the BLCs and SLCs, respectively, but only 24.32 in the cornea. Very low expression values for ABCG2 were observed in all compartments, but expression of a family member ABCC4 was found uniquely in BLCs. As already mentioned, the expression pattern of the differentiation markers was inversely related to that of stemness-associated markers. Thus for cytokeratin 3 (KRT3), the values were 14.32 in the BLCs, increasing to 37.37 and 32.28 in the SLCs and cornea, respectively, and likewise for connexin 43 (GJA1), the values were growing from 133.74 in the BLCs to 182.76 and 230.34 in the SLCs and cornea, respectively.

### GO Analysis of Differentially Expressed Genes in SLCs

**Figure 4:**
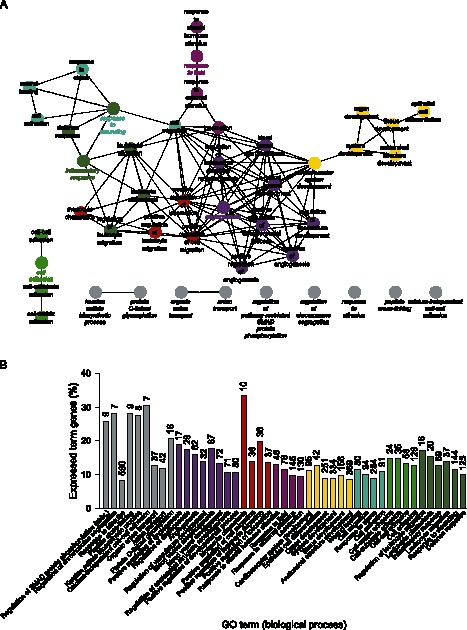
Gene ontology analysis of significantly upregulated genes in SLCs. (A) Network of significantly enriched terms. (B) Bar chart of significantly enriched terms. Numbers next to the bars indicate total number of genes mapping to the specific term.

The significant GO terms for SLCs are presented in figure 4. The SLCs are known to harbor transient accelerating cells (TACs), which are differentiating as they migrate towards the superficial central cornea. In line with the known function of this compartment to support migratory cells, we found significant GO terms for *Cell Migration* and *Locomotion.* The fact that TACs are known to differentiate and develop into mature corneal epithelium during this migration was reflected in the GO terms *Epithelial Cell Differentiation, Anatomical Structure Development, and Cell Activation.* An especially interesting finding of this analysis was the significant enrichment for terms involved in control of angiogenesis.

### Comparative BLC and SLC Pathway and GO Analyses

**Figure 5:**
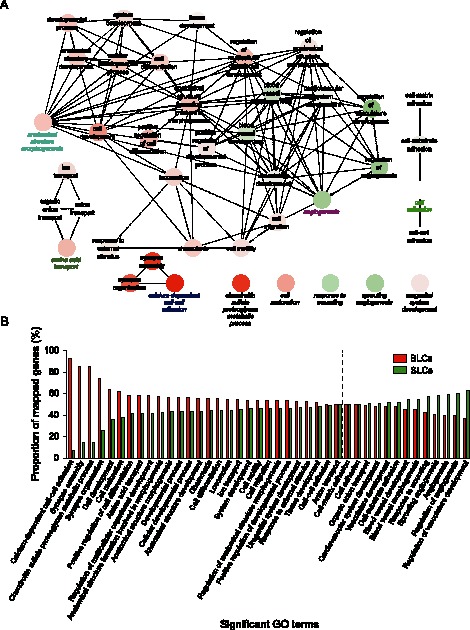
Comparative gene ontology analysis of significantly enriched terms between BLCs and SLCs. (A) Network of significantly enriched terms. (B) Bar chart of significantly enriched terms. The stippled line indicates gene equilibrium.

As evident from the comparative GO analysis, the BLCs were relatively enriched in terms related to developmental processes, synapse assembly, synapse organization, and adhesion, while SLCs were enriched for terms essentially related to vasculature (Fig. 5). The genes that were found significantly upregulated (≥2-fold, q ≤0.05) in the BLCs with respect to SLCs, were also mapped to known pathways according to KEGG. Many pathways involve the signaling and cancer-associated pathways, thus emphasizing the primitive nature of BLCs. These pathways, including their corresponding lists of mapped genes, are included in the supporting material (Table S2).

### GO Analysis of Differentially Expressed Genes in Cornea

**Figure 6:**
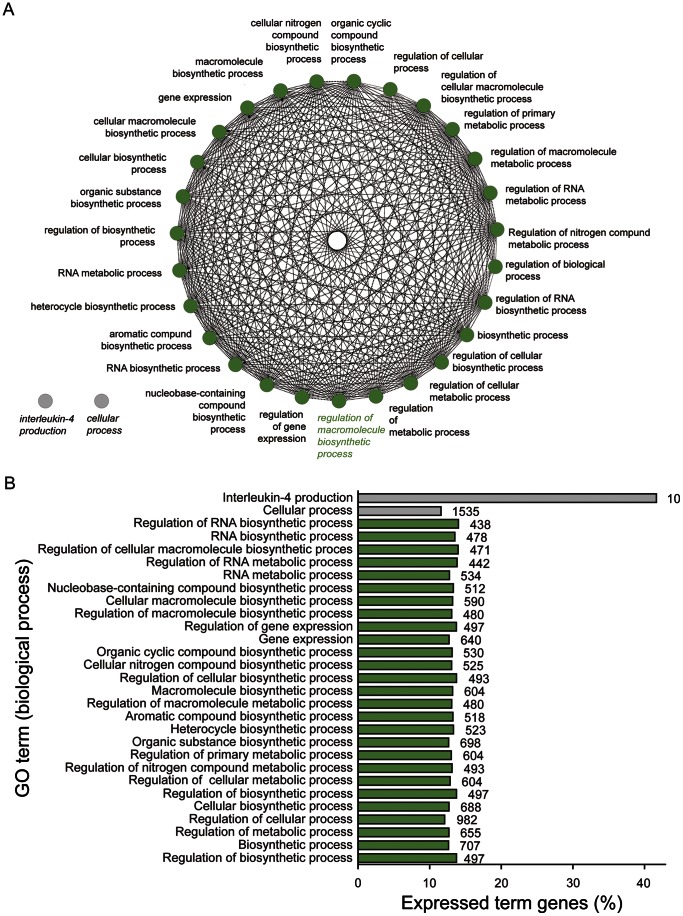
Gene ontology analysis of significantly upregulated genes in cornea. (A) Network of significantly enriched terms. (B) Bar chart of significantly enriched terms. Numbers next to the bars indicate total number of genes mapping to the specific term.

The differentiated post mitotic (PMCs) and terminally differentiated cells (TDCs) are located in paracentral/central cornea together with some late TACs. Figure 6 represents a global GO analysis of significantly enriched terms in the cornea. As expected, the biological processes in this group are of a more differentiated nature when compared with BLCs or SLCs. Most GO categories represent biosynthetic processes or regulation of biosynthetic processes. Only one large group of GO terms (>3) could be created based on the list of upregulated genes, and the leading term was *Regulation of Macromolecule Biosynthetic Process.*

### GO Analysis of Differentially Expressed Genes in Stroma

**Figure 7:**
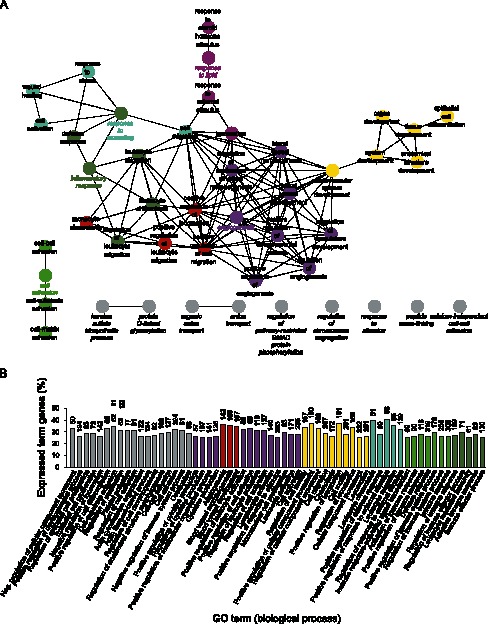
Gene ontology analysis of significantly upregulated genes in stroma. (A) Network of significantly enriched terms. (B) Bar chart of significantly enriched terms. Numbers next to the bars indicate total number of genes mapping to the specific term.

The GO analysis and network mapping for stroma is depicted in figure 7. A much more diverse group of functions was observed in this compartment than in the epithelial counterparts. Being a part of the LESC niche, it was conceivable that some molecular crosstalk between the stroma and BLCs would be revealed. Indeed, several significant GO terms related to development like *Regulation of Anatomical Structure Morphogenesis* and *Positive Regulation of Developmental Process* were identified. Additional significant terms indicating influence on epithelium included *Regulation of Epithelial Cell Proliferation,* and *Epithelial Cell Proliferation.* Other diverse functions in stroma were related to immunological function, regulation of vessel development, signaling, and cell adhesion.

### Uniquely Expressed Genes in BLCs

Using a highly powerful combination of LCM and RNA sequencing to detect novel biomarkers for LESCs, we furnish, based on the expression levels, a list of top 50 unique genes in Table 1 and a fully comprehensive list in supporting material (Table S3). As especially interesting appears the **NBL1**, which is known to be able to suppress Wnt signaling as a BMP antagonist. Thus it is plausible that it plays a role in the maintenance of LESCs. Another very interesting candidate gene is **EDAR** from the subfamily of TNFR. This gene encodes the conserved ectodysplasin A receptor, that is responsible for interactions both between mesoderm and ectoderm and within ectoderm, and is vital for normal skin embryological development [37]. The **DIDO1** is closely related to DIDO3, which has very recently been shown to be involved in stem cell development [38]. The xenobiotic transporter **ABCC4** (Multidrug resistance-associated protein 4) from the ATP-binding cassette (ABC) superfamily can also be found in this list. The function of this transporter could be of protective nature. The hallmark of stem cell compartments is tight regulation with regard to cell cycle and apoptotic events. In this context, it is not surprising that the CDC45 (cell division control protein 45 homolog) is being listed as well, along with the **CRADD** (RAIDD) that triggers apoptotic processes [39], and **PAK4,** which is known to enhance cell survival. We also found **EDNRA** in this compartment, intriguingly close to the vascular limbal network in stroma. EDNRA has been shown to interact with Tip60 (histone acetyltransferase) and HDAC7 (histone deacetylase) [40], thereby possibly affecting expression patterns within the cell.

**Table 1:** Top 50 candidate LESC markers^a^.

Ensemble ID	Gene Symbol	Description	FDR p-value	RPKM
ENSG00000143153	ATP1B1	ATPase, Na+/K+ transporting, beta 1 polypeptide	0.000449	10.25
ENSG00000158747	NBL1	neuroblastoma, suppression of tumorigenicity 1	2.75E-12	5.25
ENSG00000144057	ST6GAL2	ST6 beta-galactosamide alpha-2,6-sialyltranferase 2	0	3.33
ENSG00000111850	C6orf162	chromosome 6 open reading frame 162	0.000013	2.26
ENSG00000135960	EDAR	ectodysplasin A receptor	6.68E-14	2.21
ENSG00000113396	SLC27A6	solute carrier family 27 (fatty acid transporter), member 6	0	2.12
ENSG00000101191	DIDO1	death inducer-obliterator 1	0.03	2.07
ENSG00000256673	RP11-599J14.2	Known pseudogene	0.000415	1.94
ENSG00000183495	EP400	E1A binding protein p400	2.37E-09	1.66
ENSG00000263050	RP11-667K14.3	Novel lincRNA	0.000264	1.6
ENSG00000249742	RP11-217E13.1	Putative lincRNA	0.000264	1.49
ENSG00000143228	NUF2	NUF2, NDC80 kinetochore complex component, homolog (S. cerevisiae)	0.00000145	1.44
ENSG00000163067	ZNF2	zinc finger protein 2	4.14E-08	1.42
ENSG00000129810	SGOL1	shugoshin-like 1 (S. pombe)	0.00959	1.23
ENSG00000114790	ARHGEF26	Rho guanine nucleotide exchange factor (GEF) 26	0.000041	1.16
ENSG00000185760	KCNQ5	potassium voltage-gated channel, KQT-like subfamily, member 5	6.91E-12	1.15
ENSG00000259483	RP11-930O11.2	Novel processed transcript	0.02	1.03
ENSG00000254754	RP11-20J1.1	Putative lincRNA	0.03	0.91
ENSG00000093009	CDC45	cell division cycle 45 homolog (S. cerevisiae)	0.000084	0.89
ENSG00000169372	CRADD	CASP2 and RIPK1 domain containing adaptor with death domain	0.0021	0.85
ENSG00000260128	ULK4P2	unc-51-like kinase 4 (C. elegans) pseudogene 2	6.69E-08	0.84
ENSG00000158301	GPRASP2	G protein-coupled receptor associated sorting protein 2	0.02	0.82
ENSG00000171126	KCNG3	potassium voltage-gated channel, subfamily G, member 3	6.12E-12	0.81
ENSG00000254744	CTD-3076O17.1	Novel antisense	0.00553	0.8
ENSG00000250673	RP11-6L6.2	Putative protein coding	0.03	0.8
ENSG00000196526	AFAP1	actin filament associated protein 1	3.01E-08	0.79
ENSG00000151617	EDNRA	endothelin receptor type A	0.000825	0.76
ENSG00000125257	ABCC4	ATP-binding cassette, sub-family C (CFTR/MRP), member 4	2.68E-09	0.72
ENSG00000130669	PAK4	p21 protein (Cdc42/Rac)-activated kinase 4	0.00222	0.72
ENSG00000177098	SCN4B	sodium channel, voltage-gated, type IV, beta subunit	0.0000106	0.72
ENSG00000106665	CLIP2	CAP-GLY domain containing linker protein 2	0.02	0.71
ENSG00000112530	PACRG	PARK2 co-regulated	0.04	0.7
ENSG00000174827	PDZK1	PDZ domain containing 1	0.0000244	0.69
ENSG00000140093	SERPINA10	serpin peptidase inhibitor, clade A (alpha-1 antiproteinase, antitrypsin), member 10	0.00125	0.69
ENSG00000123473	STIL	SCL/TAL1 interrupting locus	0.000000555	0.69
ENSG00000102858	MGRN1	mahogunin ring finger 1, E3 ubiquitin protein ligase	0.00111	0.67
ENSG00000186715	MST1P9	macrophage stimulating 1 (hepatocyte growth factor-like) pseudogene 9	0.000000182	0.61
ENSG00000261777	RP11-529K1.2	Novel processed transcript	0.0000735	0.61
ENSG00000134253	TRIM45	tripartite motif containing 45	0.03	0.61
ENSG00000203872	C6orf163	chromosome 6 open reading frame 163	0.0092	0.59
ENSG00000255263	RP11-718B12.3	Putative antisense	0.00359	0.57
ENSG00000225377	RP5-1103G7.4	Novel antisense	0.01	0.57
ENSG00000215760	TAF9BP2	TAF9B RNA polymerase II, TATA box binding protein (TBP)-associated factor, 31 kDa pseudogene 2	0.05	0.57
ENSG00000158813	EDA	ectodysplasin A	0.00105	0.56
ENSG00000248125	CTB-73N10.1	Novel lincRNA	0.000974	0.55
ENSG00000236215	RP11-262H14.10	Known pseudogene	0.05	0.55
ENSG00000235167	RP11-425M5.5	Putative processed transcript	0.00854	0.54
ENSG00000095627	TDRD1	tudor domain containing 1	4.03E-11	0.54
ENSG00000125319	C17orf53	chromosome 17 open reading frame 53	0.02	0.52
ENSG00000253983	RP1-16A9.1	Novel antisense	0.02	0.51

a Genes with uniquely identified transcript in BLCs, q-value <0.05, and higher RPKM values than SLCs.

### Uniquely Expressed Genes in Cornea

Table 2 lists the top 50 genes based on RPKM values and the full list of possible differentiation markers is included in the Table S4. Of the most notable genes, the **NCK** is a member of NCK adapter protein family and has been implicated in regulation of receptor tyrosine kinases and cytoskeletal organization. **STOX1** is structurally and functionally correlated to forkhead transcription factors [41], [42], and functions as a transcription factor regulated by the PI3K-Akt pathway. Expression of STOX1 has been implicated in cell cycle control [43].

**Table 2:** Top 50 candidate differentiation markers^a^.

Ensemble ID	Gene Symbol	Description	FDR p-value	RPKM
ENSG00000137460	FHDC1	FH2 domain containing 1	1.99E-14	3.83
ENSG00000184887	BTBD6	BTB (POZ) domain containing 6	3.85E-07	3.56
ENSG00000071051	NCK2	NCK adaptor protein 2	1.60E-09	2.94
ENSG00000165730	STOX1	storkhead box 1	1.14E-03	2.26
ENSG00000169379	ARL13B	ADP-ribosylation factor-like 13B	0	2.12
ENSG00000125898	FAM110A	family with sequence similarity 110, member A	2.15E-09	2.04
ENSG00000229109	RP11-439K3.1	Putative antisense	8.69E-05	1.91
ENSG00000228470	RP11-176D17.3	Novel processed transcript	0.02	1.88
ENSG00000249955	RP11-6E9.4	Novel antisense	1.19E-03	1.82
ENSG00000162076	FLYWCH2	FLYWCH family member 2	0.04	1.66
ENSG00000263041	RP11-355F22.1	Known processed transcript	2.94E-05	1.49
ENSG00000198945	L3MBTL3	l(3)mbt-like 3 (Drosophila)	7.78E-09	1.46
ENSG00000257542	OR7E5P	olfactory receptor, family 7, subfamily E, member 5 pseudogene	1.04E-04	1.46
ENSG00000224616	RP11-305E17.6	Novel antisense	5.43E-03	1.41
ENSG00000173610	UGT2A1	UDP glucuronosyltransferase 2 family, polypeptide A1, complex locus	0	1.29
ENSG00000145864	GABRB2	gamma-aminobutyric acid (GABA) A receptor, beta 2	0	1.18
ENSG00000148300	REXO4	REX4, RNA exonuclease 4 homolog (S. cerevisiae)	2.93E-04	1.14
ENSG00000236624	CCDC163P	coiled-coil domain containing 163, pseudogene	4.52E-07	0.96
ENSG00000260548	RP6-24A23.6	Putative protein coding	3.27E-03	0.95
ENSG00000185437	SH3BGR	SH3 domain binding glutamic acid-rich protein	2.05E-03	0.95
ENSG00000120217	CD274	CD274 molecule	5.17E-12	0.93
ENSG00000089041	P2RX7	purinergic receptor P2X, ligand-gated ion channel, 7	1.57E-06	0.9
ENSG00000171428	NAT1	N-acetyltransferase 1 (arylamine N-acetyltransferase)	0.02	0.87
ENSG00000138669	PRKG2	protein kinase, cGMP-dependent, type II	1.03E-14	0.75
ENSG00000239388	ASB14	ankyrin repeat and SOCS box containing 14	5.90E-05	0.73
ENSG00000254731	CTD-2005H7.1	Novel lincRNA	0.04	0.73
ENSG00000134256	CD101	CD101 molecule	4.96E-03	0.68
ENSG00000260281	RP11-329J18.2	Novel antisense	0.01	0.65
ENSG00000197093	GAL3ST4	galactose-3-O-sulfotransferase 4	1.26E-07	0.62
ENSG00000066185	ZMYND12	zinc finger, MYND-type containing 12	1.24E-03	0.6
ENSG00000236123	CEACAMP11	carcinoembryonic antigen-related cell adhesion molecule pseudogene 11	0.03	0.59
ENSG00000050030	KIAA2022	Known protein coding	5.67E-06	0.59
ENSG00000255647	AC093510.1	Known pseudogene	0.03	0.52
ENSG00000253549	RP11-317J10.2	Novel antisense	1.59E-03	0.48
ENSG00000248371	CTC-347C20.2	Putative lincRNA	0.02	0.46
ENSG00000166321	NUDT13	nudix (nucleoside diphosphate linked moiety X)-type motif 13	0.05	0.46
ENSG00000233639	AC018730.1	Novel antisense	0.02	0.45
ENSG00000188820	FAM26F	family with sequence similarity 26, member F	3.27E-03	0.45
ENSG00000236409	NRADDP	neurotrophin receptor associated death domain, pseudogene	0.04	0.45
ENSG00000141028	CDRT15P1	CMT1A duplicated region transcript 15 pseudogene 1	0.03	0.44
ENSG00000251584	RP11-440I14.3	Putative lincRNA	0.03	0.44
ENSG00000257052	RP11-881M11.2	Novel antisense	0.03	0.43
ENSG00000183423	LRIT3	leucine-rich repeat, immunoglobulin-like and transmembrane domains 3	2.92E-04	0.41
ENSG00000152208	GRID2	glutamate receptor, ionotropic, delta 2	5.36E-14	0.4
ENSG00000253479	RP11-744J10.3	Novel lincRNA	0.05	0.4
ENSG00000182814	FUNDC2P2	FUN14 domain containing 2 pseudogene 2	6.79E-03	0.39
ENSG00000137766	UNC13C	unc-13 homolog C (C. elegans)	6.24E-14	0.39
ENSG00000184374	COLEC10	collectin sub-family member 10 (C-type lectin)	3.80E-03	0.38
ENSG00000165807	PPP1R36	protein phosphatase 1, regulatory subunit 36	1.19E-05	0.37
ENSG00000203871	C6orf164	Known protein coding	0.02	0.36

a Genes with uniquely identified transcripts in cornea, q-value <0.05, and higher RPKM values than SLCs.

## Discussion

The present investigation is, to the best of our knowledge, the first study to couple a highly expedited tissue harvest with the selectivity of laser capture and the extreme depth of massive parallel sequencing to obtain *in vivo*-relevant transcriptomic profiles of differentiation-specific corneal compartments with unprecedented resolution. Previously, attempts have been made to perform transcriptional profiling of corneal epithelium using older technology based on microarrays [44], tissue from other species [45], culture systems [46], or potentially deteriorated samples from cadavers [44], [47]. Selection of appropriate transcriptional analysis appeared central to our investigation, since the stem cells are believed to exist in niches in low-numbers as small [48] and dormant cells [19], and the rare transcripts may thus avoid detection in case of inappropriate technology. Very important was also optimization of the harvest procedure to less than five minutes, as it has been shown that the duration of cold ischemia beyond this interval results in progressively altered gene expression [49]. The location of differentiated cells in cornea, migratory activated cells in SLCs, and primitive precursor cells in BLCs was confirmed by GO analysis and lent further support for the current concept of corneal homeostasis [18].

The SLCs represent a site with major prevalence of TACs as they migrate centripetally and superficially and differentiate into PMCs and TDCs. In addition to significant GO terms related to migration and epithelial cell differentiation, we also found a plethora of significantly upregulated genes involved in angiogenesis. Among the 72 genes that mapped to vessel-related ontology terms, it is especially the flt1, which stands out. Importantly, expression of several short isoforms, which are known to be soluble, was confirmed. This finding may be an answer to a long-standing conundrum as to how the cornea maintains its avascularity and may provide further support for previously reported results [50]. This information could also partly explain angiogenic disturbances frequently observed in limbal stem cell deficiency (LSCD). Further analysis of the individual genes highlighted in our study should yield a better understanding of the mechanisms implicated in the control of limbal vasculature.

The primitive nature of the BLC compartment was evident from both KEGG mapping and upregulated GO terms. The most prominent terms related to developmental pathways and included terms such as *Cell Development* and *Negative Regulation of Canonical Wnt Receptor Signaling Pathway*. It is indeed Wnt signaling, that is recognized as a central player in stem cell activation [51]. Another very interesting finding, which could be specifically attributed to the BLCs, involved identification of highly active pigmentation processes. Only the cells in BLCs are known to acquire a pigment cap, that is presumed to protect LESCs from UV-induced damage [36]. Furthermore, our data appear to highlight a novel link between the processes underlying the development and homeostasis of BLCs. It has been suggested that during development, the neural crest-derived Sox10^+^ Schwann cell precursors (SCPs) along peripheral nerves are directed to melanocyte fate by microphtalmia-associated transcription factor (MITF) [52]. Our investigation of BLCs in the developed limbus indicates as well, high activity of processes in the categories covering *Neural Crest Cell Development* and *Neural Crest Cell Differentiation*, entailing most noteworthy up-regulation of SOX10 and MITF. It thus appears, that at least some aspects of the maintenance of cornea are governed by mechanisms that are analogous to those taking place during embryogenesis.

In addition to cell development, it was processes related to innervation that scored high in the analysis of BLCs, underscoring yet another possible dimension in the control of limbal homeostasis. Very recently, limbal innervation was shown to maintain LESCs, as these stem cells were lost in a neurotrophic keratopathy mouse model [53]. Similarly, sub basal nerve plexus was recently confirmed to be absent from human patients suffering from LSCD, as evident from *in vivo* confocal analysis [54]. Based on this evidence, one is tempted to surmise that the presence of corneal epithelial wounds could be signaled to LESCs by neural mechanisms in order to initiate the regenerative process. Taken together, the accumulated data clearly indicate that uncompromised crosstalk between LESCs and the nervous system is essential for the limbal integrity.

In an effort to identify novel LESC biomarkers, our attention was drawn to a small group of three genes including Lrig1, NFATc1, and SOX9. In the current study Lrig1 has been found specifically upregulated in the BLCs, and previously, it has been implicated in the control of epidermal stem cell quiescence [55], [56]. Assuming a role in the context of homeostasis of other tissues, the family of Lrig genes appears especially pertinent. In epidermis, Lrig1 has been recognized to play a major role, as mentioned earlier, but co-expression with other family member Lgr6 seems important as well [57]. The resemblance of epidermal expression profile, involving Lrg1 and Lgr6 but not Lgr5, with that of the BLCs, thus points to the similarity of processes involved in the regulation of these two niches. The significance of Lrig1 as a possible LESC marker is further substantiated by the recent data emphasizing its role in intestinal stem cell homeostasis [58]. Hence, we propose that Lrig1, along with other family members, should be further studied as a major *in* and *ex vivo* candidate marker. Other interesting candidates were found among the genes, which were uniquely and significantly expressed in either the stem cell or the differentiation niches. Ongoing and future work is aimed to shed more light on the significance of the proposed biomarkers.

Finally, as it has previously been shown that hypoxia supports stemness of *ex vivo* expanded stem cells [7], [59]–[61], it was of special interest to explore whether the high level of transcriptomic detail could contribute to our understanding of hypoxic regulations in the limbal niche. Analysis provided a complex picture, where the hypoxic nature of the niche was corroborated by upregulation of downstream targets for HIF-1, such as the PGF (placental growth factor), VEGFA (vascular endothelial factor A), and VEGFC (vascular endothelial factor C). On the other hand, the activation of prolylhydroxylases PHD1 and PHD2 indicated that mechanisms are in place that tend to mitigate the effect of HIF signaling. Of interest was also finding an upregulation of the pro-survival gene BCL2, which has previously been shown to enhance survival of embryonic stem cells [62]. The hypoxic networks in the limbal niche thus appear complex, nevertheless, our data confirm that oxygen is a factor, which needs to be recognized as one of the pivotal factors controlling the maintenance of stem cells.

Surprisingly, we did not find any significant ABCG2 expression in the BLCs. This membrane bound transporter is responsible for the efflux of Hoechst 33342, thereby providing for the so-called side population believed to include stem cells [15], [63]. This could be explained by either a true lack of expression in the particular cryosections [15] or that this particular transcript has escaped assembly by the employed algorithm. The first alternative seems more plausible, as the accuracy of laser capture microdissection was evident from high expression of presumed stem cell markers ΔNp63α [4], [64], [65] and cytokeratin 19 [66]–[68] and conversely low expression of presumed differentiation markers cytokeratin 3 [69] and connexin 43 [15]. Intriguingly, we did find in BLCs a high and unique expression of the family member ABCC4. Regarding the cytokeratin 19, this molecule lends itself as a convenient marker to diagnose LSCD by impression cytology [70], since it is absent in the central corneal epithelial cells [71]. Furthermore, KRT19 has previously been suggested to be a marker of epidermal stem cells [72], stem cells in the hair follicle [73], the side population of conjunctival epithelial cells [74], basal epithelial cells in corneal limbus [66]–[68], and basal cells of limbal epithelial stem cell cultures [6], [75], [76]. Recently, KRT19 was also found upregulated in limbal crypts based on microarray analysis [44]. Our own data thus appear to corroborate the previous observations.

Due to the highly stringent requirements in the tissue selection process, we were not able to include additional donors in our study, and thereby further biological replicates. We aimed to balance this limitation by using rigorous criteria for the selection of the genes. As it is beyond the scope of the current paper to provide comprehensive information on all mapped genes, ongoing work will further elucidate the role of individual biomarkers, activated pathways, and interplay between the stem cells and the surroundings. It is expected that this new information will contribute to further refinement of conditions for *ex vivo* expansion of LESCs, increase the quality of grafts prior to CLET, and at the same time enable new ways of diagnosing the LSCD.

## Supporting Information

Figure S1 **Gene ontology analysis of genes within the leading term** ***Cell Development*** **in BLCs.** (A) Network of significantly enriched terms. (B) Bar chart of significantly enriched terms. Numbers next to the bars indicate total number of genes mapping to the specific term.
(EPS)
