# Correction: Transcriptional Dissection of Human Limbal Niche Compartments by Massive Parallel Sequencing

**DOI:** 10.1371/annotation/5326b2ea-4388-4d50-8b86-407a3c5250e4

**Published:** 2013-11-08

**Authors:** Chris Bath, Danson Muttuvelu, Jeppe Emmersen, Henrik Vorum, Jesper Hjortdal, Vladimir Zachar

The Supporting Information Tables S1-S4 were erroneously omitted. Please see the missing Tables below.

Table S1: 

Table S2: 

Table S3: 

Table S4: 
