# Supplementary material for: Correction: Transcriptional Dissection of Human Limbal Niche Compartments by Massive Parallel Sequencing
Source: PLoS One. 2013 Nov 8;8(11):10.1371/annotation/5326b2ea-4388-4d50-8b86-407a3c5250e4. doi: 10.1371/annotation/5326b2ea-4388-4d50-8b86-407a3c5250e4 (PMC3826720; doi:10.1371/annotation/5326b2ea-4388-4d50-8b86-407a3c5250e4)
Supplement: Supplementary file 1 [file pone.5326b2ea-4388-4d50-8b86-407a3c5250e4.s001.docx]

| **Table S1**. Mapping statistics for fragment counting^a^. |
| --- |

|  | **Total fragments** | **Counted fragments** | **Fragments mapped** | | **Uncounted fragments** | **Total exon mapping (%)** |
| --- | --- | --- | --- | --- | --- | --- |
|  |  |  | **uniquely** | **non-uniquely** |  |  |
| **Niche** | 43,287,330 | 24,106,714 | 22,427,252 | 1,679,462 | 19,180,616 | 29.0 |
| **BLCs** | 46,355,677 | 28,298,694 | 26,388,324 | 1,910,370 | 18,056,983 | 33.3 |
| **SLCs** | 42,792,499 | 25,431,793 | 23,890,669 | 1,541,124 | 17,360,706 | 33.3 |
| **Cornea** | 53,253,266 | 31,816,985 | 29,678,078 | 2,138,907 | 21,436,281 | 35.3 |

| ^a^Intact pairs are counted as one. Broken pairs are ignored. |
| --- |
