# Supplementary material for: Correction: Transcriptional Dissection of Human Limbal Niche Compartments by Massive Parallel Sequencing
Source: PLoS One. 2013 Nov 8;8(11):10.1371/annotation/5326b2ea-4388-4d50-8b86-407a3c5250e4. doi: 10.1371/annotation/5326b2ea-4388-4d50-8b86-407a3c5250e4 (PMC3826720; doi:10.1371/annotation/5326b2ea-4388-4d50-8b86-407a3c5250e4)
Supplement: Supplementary file 2 [file pone.5326b2ea-4388-4d50-8b86-407a3c5250e4.s002.docx]

| **Table S2.** Enriched genes in BLCs mapped to KEGG pathways^a^. | | |
| --- | --- | --- |
| **KEGG pathway** | **Approved gene symbol** | **Genes/term (n)** |
| Adipocytokine signaling pathway | ACACB, ACSBG2, ACSL4, AKT3, IRS2, IRS4, LEP, NFKBIE, PCK2, PRKCQ, TRAF2 | 11 |
| Alcoholism | CREB5, GNAO1, GNG2, GNG7, HDAC11, HDAC3, HIST1H2BN, HIST1H3C, HIST1H4D, MAP2K1, NTRK2, PPP1R1B, SHC3 | 13 |
| Alzheimer's disease | APOE, APP, CDK5R1, COX4I2, LPL, NDUFA4L2, NDUFS7, NOS1, PLCB1, SNCA | 10 |
| Amoebiasis | ACTN1, ADCY1, COL4A6, COL5A2, COL5A3, CXCL1, LAMA3, LAMB2, LAMB4, PIK3R1, PLCB1, PRKACB, TGFB2 | 13 |
| Arrhythmogenic right ventricular cardiomyopathy (ARVC) | ACTN1, CACNB1, CACNB4, CACNG4, CDH2, ITGA11, LEF1, RYR2, SGCB, SGCD | 10 |
| Axon guidance | EFNA3, EPHA3, EPHB1, FYN, NCK2, NFATC1, NTN4, PAK3, PAK4, PLXNA3, PLXNC1, SEMA3C, SEMA4F, SEMA5B, UNC5C | 15 |
| Calcium signaling pathway | ADCY1, ADCY2, ADCY9, ATP2B2, BST1, CACNA1H, CHRNA7, EDNRA, EDNRB, LTB4R2, NOS1, ORAI2, ORAI3, P2RX7, PDE1C, PLCB1, PLCZ1, PRKACB, PTGFR, RYR1, RYR2 | 21 |
| Cell adhesion molecules (CAMs) | CADM1, CD274, CD40, CD8A, CDH2, CDH3, CNTN2, HLA-DPB1, JAM2, JAM3, NCAM1, NLGN1, NLGN3, NLGN4X, NRCAM, NRXN1, PVR, SDC2, SDC3 | 21 |
| Cell cycle | ANAPC2, BUB1B, CCNB1, CCND2, CCNE2, CDC14A, CDC45, CHEK1, E2F5, MCM2, MCM6, ORC6, SMC1B, TGFB2 | 14 |
| Chemokine signaling pathway | ADCY1, ADCY2, ADCY9, ADRBK2, AKT3, CCL22, CCL27, CCL28, CCR2, CXCL1, CXCL10, CXCL2, CXCL3, CXCL9, GNG2, GNG7, MAP2K1, PIK3R1, PLCB1, PRKACB, PRKCZ, PXN, SHC3, TIAM2, VAV2 | 25 |
| Cholinergic synapse | ADCY1, ADCY2, ADCY9, AKT3, BCL2, CHRNA7, CREB5, FYN, GNAO1, GNG2, GNG7, KCNQ5, MAP2K1, PIK3R1, PLCB1, PRKACB | 16 |
| Circadian entrainment | ADCY1, ADCY2, ADCY9, CACNA1H, GNAO1, GNG2, GNG7, GRIA4, NOS1, PLCB1, PRKACB, PRKG2, RYR1, RYR2 | 14 |
| Cytokine-cytokine receptor interaction | BMP2, BMPR1A, CCL22, CCL27, CCL28, CCR2, CD40, CNTF, CNTFR, CTF1, CXCL1, CXCL10, CXCL2, CXCL3, CXCL9, EDA, EDAR, GHR, IL11RA, IL17RB, IL22RA1, IL24, IL28RA, IL7, LEP, LTBR, OSMR, TGFB2, TNFRSF14, TNFRSF18, VEGFA, VEGFC | 32 |
| Dilated cardiomyopathy | ADCY1, ADCY2, ADCY9, CACNB1, CACNB4, CACNG4, ITGA11, PRKACB, RYR2, SGCB, SGCD, TGFB2 | 12 |
| Dopaminergic synapse | AKT3, CREB5, GNAO1, GNG2, GNG7, GRIA4, KIF5C, PLCB1, PPP1R1B, PPP2R5B, PPP2R5D, PRKACB | 12 |
| ECM-receptor interaction | AGRN, COL4A6, COL5A2, COL5A3, ITGA11, LAMA3, LAMB2, LAMB4, SV2B, TNC, TNN | 11 |
| Endocytosis | ADRBK2, AGAP1, CLTCL1, DNM1, FAM125B, GIT1, HSPA2, IQSEC1, PML, PRKCZ, SMAD7, SNF8, TGFB2 | 13 |
| Epstein-Barr virus infection | AKT3, BCL2, BST1, CD40, ENTPD1, HLA-DPB1, HSPA2, MAP3K14, NFKBIE, PIK3R1, POLR2I, POLR3H, PRKACB, TRAF1, TRAF2, TRAF5 | 16 |
| Focal adhesion | ACTN1, AKT3, BCL2, CCND2, COL4A6, COL5A2, COL5A3, ELK1, FLNB, FYN, ITGA11, LAMA3, LAMB2, LAMB4, MAP2K1, PAK3, PAK4, PDGFD, PGF, PIK3R1, PXN, SHC3, TNC, TNN, VAV2, VEGFA, VEGFC | 27 |
| GABAergic synapse | ADCY1, ADCY2, ADCY9, GABRB2, GABRE, GLS2, GNAO1, GNG2, GNG7, PLCL1, PRKACB | 11 |
| Gastric acid secretion | ADCY1, ADCY2, ADCY9, ATP1B2, KCNJ1, KCNJ10, KCNK10, PLCB1, PRKACB, SLC26A7 | 10 |
| Glutamatergic synapse | ADCY1, ADCY2, ADCY9, ADRBK2, GLS2, GNAO1, GNG2, GNG7, GRIA4, GRM3, PLCB1, PRKACB, SLC1A6, TRPC1 | 14 |
| Glycerophospholipid metabolism | AGPAT1, C17orf48, CRLS1, DGKD, LCAT, LPIN1, PCYT2, PLB1, PNPLA7, PTDSS2 | 10 |
| HIF-1 signaling pathway | AKT3, BCL2, EGLN2, EGLN3, ENO2, LTBR, MAP2K1, NPPA, PGF, PIK3R1, TF, TIMP1, VEGFA, VEGFC | 14 |
| HTLV-I infection | ADCY1, ADCY2, ADCY9, AKT3, ANAPC2, BUB1B, CCND2, CD3E, CD40, CHEK1, CRTC1, CRTC3, ELK1, ETS1, FZD1, FZD3, FZD7, HLA-DPB1, LTBR, MAP3K14, MRAS, NFATC1, PIK3R1, POLE2, PRKACB, TCF3, TGFB2, WNT10A | 28 |
| Hepatitis B | AKT3, BCL2, BIRC5, CCNE2, CREB5, ELK1, MAP2K1, NFATC1, PIK3R1, STAT4, TGFB2 | 11 |
| Insulin signaling pathway | ACACB, AKT3, ELK1, IRS2, IRS4, MAP2K1, PCK2, PDE3A, PDE3B, PIK3R1, PRKACB, PRKCZ, RPTOR, SHC3, SOCS2 | 15 |
| Jak-STAT signaling pathway | AKT3, CCND2, CNTF, CNTFR, CTF1, GHR, IL11RA, IL22RA1, IL24, IL28RA, IL7, LEP, OSMR, PIK3R1, SOCS2, SPRED1, SPRED2, SPRY4, STAT4 | 19 |
| MAPK signaling pathway | AKT3, CACNA1H, CACNB1, CACNB4, CACNG4, DDIT3, ELK1, FGF14, FGF2, FGFR1, FLNB, HSPA2, MAP2K1, MAP3K14, MAPK8IP1, MRAS, NTRK2, PPP5C, PRKACB, RPS6KA1, RPS6KA6, STMN1, TAOK2, TGFB2, TRAF2 | 25 |
| Measles | AKT3, CCND2, CCNE2, CD3E, FYN, HSPA2, MSN, PIK3R1, PRKCQ, TP73 | 10 |
| Melanogenesis | ADCY1, ADCY2, ADCY9, DCT, EDNRB, FZD1, FZD3, FZD7, GNAO1, LEF1, MAP2K1, MITF, PLCB1, PRKACB, TYR, TYRP1, WNT10A | 17 |
| Mineral absorption | ATP1B2, CLCN2, CYBRD1, MT1F, MT1X, MT2A, SLC26A3, SLC39A4, SLC46A1, SLC5A1, STEAP2, TF | 12 |
| Morphine addiction | ADCY1, ADCY2, ADCY9, ADRBK2, GABRB2, GABRE, GNAO1, GNG2, GNG7, PDE1C, PDE3A, PDE3B, PDE4A, PRKACB | 14 |
| NF-kappa B signaling pathway | BCL2, BCL2A1, CD40, CXCL2, LAT, LTBR, MAP3K14, PRKCQ, TRAF1, TRAF2, TRAF5 | 11 |
| Natural killer cell mediated cytotoxicity | FYN, GZMB, LAT, MAP2K1, MICB, NFATC1, PIK3R1, SH3BP2, SHC3, VAV2 | 10 |
| Neuroactive ligand-receptor interaction | ADRA2A, CHRNA2, CHRNA7, EDNRA, EDNRB, GABRB2, GABRE, GHR, GPR156, GRIA4, GRM3, LEP, LTB4R2, P2RX7, PTGDR, PTGFR | 16 |
| Neurotrophin signaling pathway | AKT3, BCL2, MAP2K1, NFKBIE, NGFRAP1, NTRK2, PIK3R1, RPS6KA1, RPS6KA6, SH2B3, SHC3, TP73 | 12 |
| Oocyte meiosis | ADCY1, ADCY2, ADCY9, ANAPC2, CCNB1, CCNE2, FBXO43, FBXO5, MAP2K1, PLCZ1, PPP2R5B, PPP2R5D, PRKACB, REC8, RPS6KA1, RPS6KA6, SMC1B, SPDYA | 18 |
| PI3K-Akt signaling pathway | AKT3, BCL2, CCND2, CCNE2, COL4A6, COL5A2, COL5A3, CREB5, EFNA3, FGF14, FGF2, FGFR1, GHR, GNG2, GNG7, IL7, ITGA11, LAMA3, LAMB2, LAMB4, MAP2K1, OSMR, PCK2, PDGFD, PGF, PHLPP2, PIK3R1, PPP2R5B, PPP2R5D, PRKCZ, RPTOR, TNC, TNN, VEGFA, VEGFC | 35 |
| Pancreatic secretion | ADCY1, ADCY2, ADCY9, ATP1B2, ATP2B2, BST1, PLCB1, RYR2, SLC26A3, TRPC1 | 10 |
| Pathways in cancer | AKT3, AXIN2, BCL2, BIRC5, BMP2, CCNE2, COL4A6, EGLN2, EGLN3, ETS1, FGF14, FGF2, FGFR1, FZD1, FZD3, FZD7, GLI3, LAMA3, LAMB2, LAMB4, LEF1, MAP2K1, MITF, NKX3-1, PAX8, PGF, PIK3R1, PML, RAD51, RARA, TGFB2, TRAF1, TRAF2, TRAF5, VEGFA, VEGFC, WNT10A | 37 |
| Progesterone-mediated oocyte maturation | ADCY1, ADCY2, ADCY9, AKT3, ANAPC2, CCNB1, MAP2K1, PDE3A, PDE3B, PIK3R1, PRKACB, RPS6KA1, RPS6KA6, SPDYA | 14 |
| Prostate cancer | AKT3, BCL2, CCNE2, CREB5, FGFR1, LEF1, MAP2K1, NKX3-1, PDGFD, PIK3R1 | 10 |
| Purine metabolism | ADCY1, ADCY2, ADCY9, AK4, AK5, C17orf48, ENPP1, ENTPD1, GMPR, GUCY2C, HPRT1, NME4, NT5M, PDE1C, PDE3A, PDE3B, PDE4A, PGM1, POLE2, POLR2I, POLR3H | 21 |
| Regulation of actin cytoskeleton | ABI2, ACTN1, ENAH, FGF14, FGF2, FGFR1, GIT1, ITGA11, MAP2K1, MRAS, MSN, PAK3, PAK4, PDGFD, PIK3R1, PXN, TIAM2, VAV2 | 18 |
| Renal cell carcinoma | AKT3, EGLN2, EGLN3, ETS1, FLCN, MAP2K1, PAK3, PAK4, PGF, PIK3R1, TGFB2, VEGFA, VEGFC | 13 |
| Retrograde endocannabinoid signaling | ADCY1, ADCY2, ADCY9, GABRB2, GABRE, GNAO1, GNG2, GNG7, GRIA4, PLCB1, PRKACB | 10 |
| Salivary secretion | ADCY1, ADCY2, ADCY9, ATP1B2, ATP2B2, BST1, NOS1, PLCB1, PRKACB, PRKG2, VAMP2 | 11 |
| Serotonergic synapse | ALOX12, APP, CYP4X1, GABRB2, GNAO1, GNG2, GNG7, KCNN2, MAP2K1, PLCB1, PRKACB, SLC6A4, TRPC1 | 13 |
| Small cell lung cancer | AKT3, BCL2, CCNE2, COL4A6, LAMA3, LAMB2, LAMB4, PIK3R1, TRAF1, TRAF2, TRAF5 | 11 |
| T cell receptor signaling pathway | AKT3, CD3E, CD8A, FYN, LAT, MAP2K1, MAP3K14, NCK2, NFATC1, NFKBIE, PAK3, PAK4, PIK3R1, PRKCQ, VAV2 | 15 |
| Tight junction | ACTN1, AKT3, EPB41L2, HCLS1, JAM2, JAM3, MRAS, MYH3, PRKCQ, PRKCZ | 10 |
| Toxoplasmosis | AKT3, BCL2, CD40, GNAO1, HLA-DPB1, HSPA2, LAMA3, LAMB2, LAMB4, PIK3R1, TGFB2 | 11 |
| Transcriptional misregulation in cancer | BCL2A1, CCND2, CD40, DDIT3, DOT1L, ETV5, EYA1, FUS, FUT8, GZMB, HIST1H3C, MLLT3, PAX8, PML, PROM1, RARA, RUNX2, TCF3, TRAF1 | 19 |
| Ubiquitin mediated proteolysis | ANAPC2, FANCL, FBXO2, KEAP1, MGRN1, PML, SIAH1, UBE2M, UBE2O, UBE2QL1, UBE2S | 11 |
| Vascular smooth muscle contraction | ADCY1, ADCY2, ADCY9, CALD1, EDNRA, KCNMB2, MAP2K1, MYL6B, PLCB1, PRKACB, PRKCQ | 11 |
| Viral carcinogenesis | ACTN1, CCND2, CCNE2, CHEK1, CREB5, GTF2A1L, GTF2H2, HDAC11, HDAC3, HIST1H2BN, HIST1H4D, LTBR, PIK3R1, PRKACB, PXN, TRAF1, TRAF2, TRAF5 | 18 |
| Wnt signaling pathway | AXIN2, CCND2, FZD1, FZD3, FZD7, GPC4, LEF1, LRP5, NFATC1, PLCB1, PPP2R5B, PPP2R5D, PRICKLE2, PRKACB, SFRP1, SIAH1, WNT10A | 17 |

^a^Gene set is defined by FDR < 0.05 and upregulation ≥ 2-fold compared to SLCs.
