# Supplementary material for: Correction: Transcriptional Dissection of Human Limbal Niche Compartments by Massive Parallel Sequencing
Source: PLoS One. 2013 Nov 8;8(11):10.1371/annotation/5326b2ea-4388-4d50-8b86-407a3c5250e4. doi: 10.1371/annotation/5326b2ea-4388-4d50-8b86-407a3c5250e4 (PMC3826720; doi:10.1371/annotation/5326b2ea-4388-4d50-8b86-407a3c5250e4)
Supplement: Supplementary file 3 [file pone.5326b2ea-4388-4d50-8b86-407a3c5250e4.s003.docx]

| Table S3. Candidate biomarkers for LESCs sorted alphabetically based on gene symbol^a^. | | | | |
| --- | --- | --- | --- | --- |
| Ensemble ID | **Gene Symbol** | **Description** | **FDR p-value** | **RPKM** |
| ENSG00000256069 | A2MP1 | alpha-2-macroglobulin pseudogene 1 | 1.03E-7 | 0.29 |
| ENSG00000125257 | ABCC4 | ATP-binding cassette, sub-family C (CFTR/MRP), member 4 | 2.68E-9 | 0.72 |
| ENSG00000236432 | AC097662.2 | Novel antisense | 8.54E-3 | 0.13 |
| ENSG00000231304 | AC107622.1 | Novel lincRNA | 2.10E-3 | 0.48 |
| ENSG00000224479 | AC136289.1 | Known pseudogene | 4.09E-4 | 0.22 |
| ENSG00000142920 | ADC | arginine decarboxylase | 2.87E-4 | 0.18 |
| ENSG00000235888 | AF064858.8 | Novel lincRNA | 0.05 | 0.20 |
| ENSG00000196526 | AFAP1 | actin filament associated protein 1 | 3.01E-8 | 0.79 |
| ENSG00000211454 | AKR7L | aldo-keto reductase family 7-like | 0.03 | 0.11 |
| ENSG00000108839 | ALOX12 | arachidonate 12-lipoxygenase | 0.05 | 0.15 |
| ENSG00000198720 | ANKRD13B | ankyrin repeat domain 13B | 1.50E-3 | 0.14 |
| ENSG00000226435 | ANKRD18DP | ankyrin repeat domain 18D, pseudogene | 3.59E-3 | 0.14 |
| ENSG00000114790 | ARHGEF26 | Rho guanine nucleotide exchange factor (GEF) 26 | 4.10E-5 | 1.16 |
| ENSG00000143153 | ATP1B1 | ATPase, Na+/K+ transporting, beta 1 polypeptide | 4.49E-4 | 10.25 |
| ENSG00000204195 | AWAT1 | acyl-CoA wax alcohol acyltransferase 1 | 6.32E-4 | 0.44 |
| ENSG00000160862 | AZGP1 | alpha-2-glycoprotein 1, zinc-binding | 3.57E-3 | 0.22 |
| ENSG00000141577 | AZI1 | 5-azacytidine induced 1 | 0.02 | 0.28 |
| ENSG00000182272 | B4GALNT4 | beta-1,4-N-acetyl-galactosaminyl transferase 4 | 8.39E-3 | 0.14 |
| ENSG00000171282 | BAHCC1 | BAH domain and coiled-coil containing 1 | 8.65E-6 | 0.14 |
| ENSG00000140379 | BCL2A1 | BCL2-related protein A1 | 8.54E-3 | 0.39 |
| ENSG00000102010 | BMX | BMX non-receptor tyrosine kinase | 2.82E-3 | 0.31 |
| ENSG00000005379 | BZRAP1 | benzodiazapine receptor (peripheral) associated protein 1 | 4.68E-3 | 0.25 |
| ENSG00000110427 | C11orf41 | KIAA1549-like | 1.74E-14 | 0.48 |
| ENSG00000125319 | C17orf53 | chromosome 17 open reading frame 53 | 0.02 | 0.52 |
| ENSG00000232560 | C21orf37 | chromosome 21 open reading frame 37 | 0.03 | 0.45 |
| ENSG00000111850 | C6orf162 | chromosome 6 open reading frame 162 | 1.30E-5 | 2.26 |
| ENSG00000203872 | C6orf163 | chromosome 6 open reading frame 163 | 9.20E-3 | 0.59 |
| ENSG00000196557 | CACNA1H | calcium channel, voltage-dependent, T type, alpha 1H subunit | 0.01 | 0.06 |
| ENSG00000203697 | CAPN8 | calpain 8 | 2.32E-3 | 0.10 |
| ENSG00000103021 | CCDC113 | coiled-coil domain containing 113 | 4.81E-3 | 0.27 |
| ENSG00000135083 | CCNJL | cyclin J-like | 1.17E-8 | 0.32 |
| ENSG00000158477 | CD1A | CD1a molecule | 8.54E-3 | 0.20 |
| ENSG00000158481 | CD1C | CD1c molecule | 5.53E-3 | 0.32 |
| ENSG00000093009 | CDC45 | cell division cycle 45 homolog (S. cerevisiae) | 8.40E-5 | 0.89 |
| ENSG00000140488 | CELF6 | CUGBP, Elav-like family member 6 | 2.53E-7 | 0.38 |
| ENSG00000205923 | CEMP1 | cementum protein 1 | 0.05 | 0.15 |
| ENSG00000123219 | CENPK | centromere protein K | 7.64E-7 | 0.47 |
| ENSG00000120903 | CHRNA2 | cholinergic receptor, nicotinic, alpha 2 (neuronal) | 1.50E-3 | 0.12 |
| ENSG00000106665 | CLIP2 | CAP-GLY domain containing linker protein 2 | 0.02 | 0.71 |
| ENSG00000119946 | CNNM1 | cyclin M1 | 2.71E-7 | 0.46 |
| ENSG00000122756 | CNTFR | ciliary neurotrophic factor receptor | 1.50E-3 | 0.27 |
| ENSG00000100473 | COCH | coagulation factor C homolog, cochlin (Limulus polyphemus) | 8.35E-5 | 0.44 |
| ENSG00000206561 | COLQ | collagen-like tail subunit (single strand of homotrimer) of asymmetric acetylcholinesterase | 0.01 | 0.28 |
| ENSG00000169372 | CRADD | CASP2 and RIPK1 domain containing adaptor with death domain | 2.10E-3 | 0.85 |
| ENSG00000248125 | CTB-73N10.1 | Novel lincRNA | 9.74E-4 | 0.55 |
| ENSG00000248671 | CTD-2313N18.5 | Novel processed transcript | 8.54E-3 | 0.12 |
| ENSG00000258766 | CTD-2540C19.1 | DIO2 antisense RNA 1 | 6.32E-4 | 0.40 |
| ENSG00000259724 | CTD-2643K12.3 | Novel lincRNA | 00.03 | 00.10 |
| ENSG00000254744 | CTD-3076O17.1 | Novel antisense | 5.53E-3 | 0.80 |
| ENSG00000138755 | CXCL9 | chemokine (C-X-C motif) ligand 9 | 8.54E-3 | 0.15 |
| ENSG00000160870 | CYP3A7 | cytochrome P450, family 3, subfamily A, polypeptide 7 | 8.65E-6 | 0.44 |
| ENSG00000163357 | DCST1 | DC-STAMP domain containing 1 | 9.80E-4 | 0.22 |
| ENSG00000104808 | DHDH | dihydrodiol dehydrogenase (dimeric) | 0.02 | 0.33 |
| ENSG00000101191 | DIDO1 | death inducer-obliterator 1 | 0.03 | 2.07 |
| ENSG00000211452 | DIO1 | deiodinase, iodothyronine, type I | 9.74E-4 | 0.26 |
| ENSG00000184911 | DMRTC1B | DMRT-like family C1B | 0.02 | 0.18 |
| ENSG00000007174 | DNAH9 | dynein, axonemal, heavy chain 9 | 0.03 | 0.07 |
| ENSG00000178401 | DNAJC22 | DnaJ (Hsp40) homolog, subfamily C, member 22 | 0.04 | 0.20 |
| ENSG00000158813 | EDA | ectodysplasin A | 1.05E-3 | 0.56 |
| ENSG00000135960 | EDAR | ectodysplasin A receptor | 6.68E-14 | 2.21 |
| ENSG00000151617 | EDNRA | endothelin receptor type A | 8.25E-4 | 0.76 |
| ENSG00000154920 | EME1 | essential meiotic endonuclease 1 homolog 1 (S. pombe) | 1.33E-3 | 0.24 |
| ENSG00000260754 | EMR4P | egf-like module containing, mucin-like, hormone receptor-like 4 pseudogene | 0.02 | 0.12 |
| ENSG00000183495 | EP400 | E1A binding protein p400 | 2.37E-9 | 1.66 |
| ENSG00000121053 | EPX | eosinophil peroxidase | 5.53E-3 | 0.17 |
| ENSG00000186871 | ERCC6L | excision repair cross-complementing rodent repair deficiency, complementation group 6-like | 8.54E-3 | 0.10 |
| ENSG00000182230 | FAM153B | family with sequence similarity 153, member B | 1.50E-3 | 0.06 |
| ENSG00000146267 | FAXC | failed axon connections homolog (Drosophila) | 1.50E-3 | 0.26 |
| ENSG00000156509 | FBXO43 | F-box protein 43 | 0.05 | 0.07 |
| ENSG00000086205 | FOLH1 | folate hydrolase (prostate-specific membrane antigen) 1 | 0.03 | 0.28 |
| ENSG00000137270 | GCM1 | glial cells missing homolog 1 (Drosophila) | 0.03 | 0.12 |
| ENSG00000124194 | GDAP1L1 | ganglioside induced differentiation associated protein 1-like 1 | 3.59E-3 | 0.18 |
| ENSG00000175697 | GPR156 | G protein-coupled receptor 156 | 0.01 | 0.06 |
| ENSG00000158301 | GPRASP2 | G protein-coupled receptor associated sorting protein 2 | 0.02 | 0.82 |
| ENSG00000152578 | GRIA4 | glutamate receptor, ionotropic, AMPA 4 | 7.47E-4 | 0.20 |
| ENSG00000242441 | GTF2A1L | general transcription factor IIA, 1-like | 0.02 | 0.17 |
| ENSG00000056998 | GYG2 | glycogenin 2 | 4.83E-8 | 0.48 |
| ENSG00000127124 | HIVEP3 | human immunodeficiency virus type I enhancer binding protein 3 | 2.98E-9 | 0.31 |
| ENSG00000160207 | HSF2BP | heat shock transcription factor 2 binding protein | 8.54E-3 | 0.22 |
| ENSG00000231871 | IPO9-AS1 | IPO9 antisense RNA 1 | 0.01 | 0.31 |
| ENSG00000250588 | IQCJ-SCHIP1 | IQCJ-SCHIP1 readthrough | 1.22E-8 | 0.39 |
| ENSG00000171126 | KCNG3 | potassium voltage-gated channel, subfamily G, member 3 | 6.12E-12 | 0.81 |
| ENSG00000185760 | KCNQ5 | potassium voltage-gated channel, KQT-like subfamily, member 5 | 6.91E-12 | 1.15 |
| ENSG00000213658 | LAT | linker for activation of T cells | 7.07E-3 | 0.28 |
| ENSG00000145826 | LECT2 | leukocyte cell-derived chemotaxin 2 | 0..01 | 0.18 |
| ENSG00000213906 | LTB4R2 | leukotriene B4 receptor 2 | 1.10E-4 | 0.36 |
| ENSG00000187123 | LYPD6 | LY6/PLAUR domain containing 6 | 1.30E-12 | 0.49 |
| ENSG00000139915 | MDGA2 | MAM domain containing glycosylphosphatidylinositol anchor 2 | 4.62E-5 | 0.10 |
| ENSG00000117122 | MFAP2 | microfibrillar-associated protein 2 | 0.05 | 0.13 |
| ENSG00000102858 | MGRN1 | mahogunin ring finger 1, E3 ubiquitin protein ligase | 1.11E-3 | 0.67 |
| ENSG00000186732 | MPPED1 | metallophosphoesterase domain containing 1 | 5.66E-4 | 0.21 |
| ENSG00000186715 | MST1P9 | macrophage stimulating 1 (hepatocyte growth factor-like) pseudogene 9 | 1.82E-7 | 0.61 |
| ENSG00000158747 | NBL1 | neuroblastoma, suppression of tumorigenicity 1 | 2.75E-12 | 5.25 |
| ENSG00000175206 | NPPA | natriuretic peptide A | 0.03 | 0.28 |
| ENSG00000205309 | NT5M | 5',3'-nucleotidase, mitochondrial | 6.32E-4 | 0.32 |
| ENSG00000143228 | NUF2 | NUF2, NDC80 kinetochore complex component, homolog (S. cerevisiae) | 1.45E-6 | 1.44 |
| ENSG00000104044 | OCA2 | oculocutaneous albinism II | 1.82E-4 | 0.45 |
| ENSG00000138315 | OIT3 | oncoprotein induced transcript 3 | 0.02 | 0.15 |
| ENSG00000112530 | PACRG | PARK2 co-regulated | 0.04 | 0.70 |
| ENSG00000130669 | PAK4 | p21 protein (Cdc42/Rac)-activated kinase 4 | 2.22E-3 | 0.72 |
| ENSG00000102290 | PCDH11X | protocadherin 11 X-linked | 2.68E-4 | 0.07 |
| ENSG00000174827 | PDZK1 | PDZ domain containing 1 | 2.44E-5 | 0.69 |
| ENSG00000005421 | PON1 | paraoxonase 1 | 2.64E-4 | 0.24 |
| ENSG00000143847 | PPFIA4 | protein tyrosine phosphatase, receptor type, f polypeptide (PTPRF), interacting protein (liprin), alpha 4 | 3.35E-3 | 0.12 |
| ENSG00000212123 | PRR22 | proline rich 22 | 0.02 | 0.25 |
| ENSG00000185379 | RAD51D | RAD51 homolog D (S. cerevisiae) | 0.03 | 0.26 |
| ENSG00000167550 | RHEBL1 | Ras homolog enriched in brain like 1 | 0.01 | 0.47 |
| ENSG00000141576 | RNF157 | ring finger protein 157 | 3.59E-3 | 0.10 |
| ENSG00000253983 | RP1-16A9.1 | Novel antisense | 0.02 | 0.51 |
| ENSG00000227579 | RP1-35C21.2 | Novel lincRNA | 5.53E-3 | 0.49 |
| ENSG00000259488 | RP11-154J22.1 | Novel antisense | 8.54E-3 | 0.39 |
| ENSG00000251396 | RP11-163N6.2 | Novel processed transcript | 0.05 | 0.06 |
| ENSG00000234763 | RP11-203H2.2 | Novel lincRNA | 0.02 | 0.47 |
| ENSG00000254754 | RP11-20J1.1 | Putative lincRNA | 0.03 | 0.91 |
| ENSG00000249742 | RP11-217E13.1 | Putative lincRNA | 2.64E-4 | 1.49 |
| ENSG00000254813 | RP11-252C15.1 | Novel lincRNA | 0.05 | 0.22 |
| ENSG00000236215 | RP11-262H14.10 | Known pseudogene | 0.05 | 0.55 |
| ENSG00000180712 | RP11-290F5.2 | Novel lincRNA | 8.10E-6 | 0.42 |
| ENSG00000239569 | RP11-325F22.4 | Putative antisense | 0.03 | 0.43 |
| ENSG00000231937 | RP11-329E24.6 | Novel antisense | 0.05 | 0.51 |
| ENSG00000237101 | RP11-365O16.6 | Novel antisense | 0.03 | 0.24 |
| ENSG00000254456 | RP11-405K6.1 | Novel lincRNA | 0.02 | 0.36 |
| ENSG00000235167 | RP11-425M5.5 | Putative processed transcript | 8.54E-3 | 0.54 |
| ENSG00000238280 | RP11-436D10.3 | Novel antisense | 0.02 | 0.51 |
| ENSG00000261777 | RP11-529K1.2 | Novel processed transcript | 7.35E-5 | 0.61 |
| ENSG00000256673 | RP11-599J14.2 | Known pseudogene | 4.15E-4 | 1.94 |
| ENSG00000240602 | RP11-64D22.2 | Known pseudogene | 2.32E-3 | 0.26 |
| ENSG00000263050 | RP11-667K14.3 | Novel lincRNA | 2.64E-4 | 1.60 |
| ENSG00000250673 | RP11-6L6.2 | Putative protein coding | 0.03 | 0.80 |
| ENSG00000255263 | RP11-718B12.3 | Putative antisense | 3.59E-3 | 0.57 |
| ENSG00000226476 | RP11-776H12.1 | Novel lincRNA | 0.03 | 0.18 |
| ENSG00000255929 | RP11-867G2.8 | Novel antisense | 0.03 | 0.36 |
| ENSG00000235565 | RP11-86H7.7 | Novel lincRNA | 0.02 | 0.16 |
| ENSG00000246985 | RP11-887P2.1 | Novel lincRNA | 0..05 | 0.07 |
| ENSG00000259483 | RP11-930O11.2 | Novel processed transcript | 0.02 | 1.03 |
| ENSG00000226133 | RP4-683M8.2 | Novel lincRNA | 1.50E-3 | 0.51 |
| ENSG00000204934 | RP4-751H13.6 | ATP6V0E2 antisense RNA 1 | 0.01 | 0.12 |
| ENSG00000225377 | RP5-1103G7.4 | Novel antisense | 0.01 | 0.57 |
| ENSG00000231768 | RP5-855F14.1 | Novel lincRNA | 6.32E-4 | 0.22 |
| ENSG00000181031 | RPH3AL | rabphilin 3A-like (without C2 domains) | 1.70E-4 | 0.12 |
| ENSG00000177098 | SCN4B | sodium channel, voltage-gated, type IV, beta subunit | 1.06E-5 | 0.72 |
| ENSG00000140093 | SERPINA10 | serpin peptidase inhibitor, clade A (alpha-1 antiproteinase, antitrypsin), member 10 | 1.25E-3 | 0.69 |
| ENSG00000129810 | SGOL1 | shugoshin-like 1 (S. pombe) | 9.59E-3 | 1.23 |
| ENSG00000113396 | SLC27A6 | solute carrier family 27 (fatty acid transporter), member 6 | 0.00 | 2.12 |
| ENSG00000164175 | SLC45A2 | solute carrier family 45, member 2 | 5.93E-6 | 0.43 |
| ENSG00000100170 | SLC5A1 | solute carrier family 5 (sodium/glucose cotransporter), member 1 | 0.05 | 0.07 |
| ENSG00000117834 | SLC5A9 | solute carrier family 5 (sodium/glucose cotransporter), member 9 | 5.71E-5 | 0.29 |
| ENSG00000135740 | SLC9A5 | solute carrier family 9, subfamily A (NHE5, cation proton antiporter 5), member 5 | 4.09E-4 | 0.12 |
| ENSG00000205045 | SLFN12L | schlafen family member 12-like | 0.05 | 0.07 |
| ENSG00000158480 | SPATA2 | spermatogenesis associated 2 | 0.02 | 0.48 |
| ENSG00000144057 | ST6GAL2 | ST6 beta-galactosamide alpha-2,6-sialyltranferase 2 | 0.00 | 3.33 |
| ENSG00000123473 | STIL | SCL/TAL1 interrupting locus | 5.55E-7 | 0.69 |
| ENSG00000215760 | TAF9BP2 | TAF9B RNA polymerase II, TATA box binding protein (TBP)-associated factor, 31kDa pseudogene 2 | 0.05 | 0.57 |
| ENSG00000095627 | TDRD1 | tudor domain containing 1 | 4.03E-11 | 0.54 |
| ENSG00000170925 | TEX13B | testis expressed 13B | 0.02 | 0.28 |
| ENSG00000118707 | TGIF2 | TGFB-induced factor homeobox 2 | 1.03E-3 | 0.28 |
| ENSG00000133069 | TMCC2 | transmembrane and coiled-coil domain family 2 | 0.01 | 0.17 |
| ENSG00000137103 | TMEM8B | transmembrane protein 8B | 2.28E-7 | 0.39 |
| ENSG00000134253 | TRIM45 | tripartite motif containing 45 | 0.03 | 0.61 |
| ENSG00000119283 | TRIM67 | tripartite motif containing 67 | 3.59E-3 | 0.05 |
| ENSG00000141540 | TTYH2 | tweety homolog 2 (Drosophila) | 4.04E-6 | 0.36 |
| ENSG00000260128 | ULK4P2 | unc-51-like kinase 4 (C. elegans) pseudogene 2 | 6.69E-8 | 0.84 |
| ENSG00000144406 | UNC80 | unc-80 homolog (C. elegans) | 2.32E-3 | 0.03 |
| ENSG00000197748 | WDR96 | WD repeat domain 96 | 0.03 | 0.37 |
| ENSG00000178150 | ZNF114 | zinc finger protein 114 | 0.02 | 0.16 |
| ENSG00000163067 | ZNF2 | zinc finger protein 2 | 4.14E-8 | 1.42 |
| ^a^Genes with uniquely identified transcripts in BLCs, q-value < 0.05, and higher RPKM values than SLCs. | | | | |
