# Supplementary material for: Correction: Transcriptional Dissection of Human Limbal Niche Compartments by Massive Parallel Sequencing
Source: PLoS One. 2013 Nov 8;8(11):10.1371/annotation/5326b2ea-4388-4d50-8b86-407a3c5250e4. doi: 10.1371/annotation/5326b2ea-4388-4d50-8b86-407a3c5250e4 (PMC3826720; doi:10.1371/annotation/5326b2ea-4388-4d50-8b86-407a3c5250e4)
Supplement: Supplementary file 4 [file pone.5326b2ea-4388-4d50-8b86-407a3c5250e4.s004.docx]

| Table S4. Candidate biomarkers for differentiated LECs sorted alphabetically based on gene symbol^a^. | | | | |
| --- | --- | --- | --- | --- |
| Ensemble ID | **Gene Symbol** | **Description** | **FDR p-value** | **RPKM** |
| ENSG00000226747 | AC007966.1 | Novel lincRNA | 0.04 | 0.21 |
| ENSG00000161103 | AC008103.5 | Novel processed transcript | 1.10E-3 | 0.10 |
| ENSG00000233639 | AC018730.1 | Novel antisense | 0.02 | 0.45 |
| ENSG00000255647 | AC093510.1 | Known pseudogene | 0.03 | 0.52 |
| ENSG00000144218 | AFF3 | AF4/FMR2 family, member 3 | 7.89E-7 | 0.16 |
| ENSG00000136872 | ALDOB | aldolase B, fructose-bisphosphate | 5.32E-4 | 0.28 |
| ENSG00000171714 | ANO5 | anoctamin 5 | 0.01 | 0.06 |
| ENSG00000128805 | ARHGAP22 | Rho GTPase activating protein 22 | 0.02 | 0.15 |
| ENSG00000169379 | ARL13B | ADP-ribosylation factor-like 13B | 0.00 | 2.12 |
| ENSG00000239388 | ASB14 | ankyrin repeat and SOCS box containing 14 | 5.90E-5 | 0.73 |
| ENSG00000206190 | ATP10A | ATPase, class V, type 10A | 1.13E-4 | 0.13 |
| ENSG00000184887 | BTBD6 | BTB (POZ) domain containing 6 | 3.85E-7 | 3.56 |
| ENSG00000160392 | C19orf47 | Known protein coding | 0.04 | 0.31 |
| ENSG00000138813 | C4orf17 | Known protein coding | 3.71E-4 | 0.22 |
| ENSG00000203871 | C6orf164 | Known protein coding | 0.02 | 0.36 |
| ENSG00000036828 | CASR | calcium-sensing receptor | 0.01 | 0.08 |
| ENSG00000203799 | CCDC162P | coiled-coil domain containing 162, pseudogene | 3.05E-4 | 0.22 |
| ENSG00000236624 | CCDC163P | coiled-coil domain containing 163, pseudogene | 4.52E-7 | 0.96 |
| ENSG00000134256 | CD101 | CD101 molecule | 4.96E-3 | 0.68 |
| ENSG00000120217 | CD274 | CD274 molecule | 5.17E-12 | 0.93 |
| ENSG00000141028 | CDRT15P1 | CMT1A duplicated region transcript 15 pseudogene 1 | 0.03 | 0.44 |
| ENSG00000236123 | CEACAMP11 | carcinoembryonic antigen-related cell adhesion molecule pseudogene 11 | 0.03 | 0.59 |
| ENSG00000184293 | CLECL1 | C-type lectin-like 1 | 0.03 | 0.20 |
| ENSG00000149972 | CNTN5 | contactin 5 | 0.03 | 0.04 |
| ENSG00000171502 | COL24A1 | collagen, type XXIV, alpha 1 | 0.02 | 0.12 |
| ENSG00000230524 | COL6A4P1 | collagen, type VI, alpha 4 pseudogene 1 | 9.75E-3 | 0.10 |
| ENSG00000184374 | COLEC10 | collectin sub-family member 10 (C-type lectin) | 3.80E-3 | 0.38 |
| ENSG00000170165 | CR848007.2 | Known pseudogene | 5.43E-3 | 0.35 |
| ENSG00000248371 | CTC-347C20.2 | Putative lincRNA | 0.02 | 0.46 |
| ENSG00000254731 | CTD-2005H7.1 | Novel lincRNA | 0.04 | 0.73 |
| ENSG00000205913 | CTD-2270P14.3 | SRRM2 antisense RNA 1 | 5.32E-4 | 0.12 |
| ENSG00000183230 | CTNNA3 | catenin (cadherin-associated protein), alpha 3 | 0.04 | 0.07 |
| ENSG00000205944 | DAZ2 | deleted in azoospermia 2 | 6.47E-4 | 0.23 |
| ENSG00000062282 | DGAT2 | diacylglycerol O-acyltransferase 2 | 2.28E-3 | 0.23 |
| ENSG00000130226 | DPP6 | dipeptidyl-peptidase 6 | 0.02 | 0.03 |
| ENSG00000126860 | EVI2A | ecotropic viral integration site 2A | 0.04 | 0.10 |
| ENSG00000125898 | FAM110A | family with sequence similarity 110, member A | 2.15E-9 | 2.04 |
| ENSG00000188820 | FAM26F | family with sequence similarity 26, member F | 3.27E-3 | 0.45 |
| ENSG00000137460 | FHDC1 | FH2 domain containing 1 | 1.99E-14 | 3.83 |
| ENSG00000162076 | FLYWCH2 | FLYWCH family member 2 | 0.04 | 1.66 |
| ENSG00000182814 | FUNDC2P2 | FUN14 domain containing 2 pseudogene 2 | 6.79E-3 | 0.39 |
| ENSG00000145864 | GABRB2 | gamma-aminobutyric acid (GABA) A receptor, beta 2 | 0.00 | 1.18 |
| ENSG00000197093 | GAL3ST4 | galactose-3-O-sulfotransferase 4 | 1.26E-7 | 0.62 |
| ENSG00000187210 | GCNT1 | glucosaminyl (N-acetyl) transferase 1, core 2 | 8.99E-4 | 0.12 |
| ENSG00000152208 | GRID2 | glutamate receptor, ionotropic, delta 2 | 5.36E-14 | 0.40 |
| ENSG00000134240 | HMGCS2 | 3-hydroxy-3-methylglutaryl-CoA synthase 2 (mitochondrial) | 2.58E-4 | 0.29 |
| ENSG00000170091 | HMP19 | Neuron-specific protein family member 2 | 6.79E-3 | 0.12 |
| ENSG00000143473 | KCNH1 | potassium voltage-gated channel, subfamily H (eag-related), member 1 | 0.01 | 0.13 |
| ENSG00000050030 | KIAA2022 | Known protein coding | 5.67E-6 | 0.59 |
| ENSG00000198945 | L3MBTL3 | l(3)mbt-like 3 (Drosophila) | 7.78E-9 | 1.46 |
| ENSG00000086730 | LAT2 | linker for activation of T cells family, member 2 | 0.03 | 0.08 |
| ENSG00000183423 | LRIT3 | leucine-rich repeat, immunoglobulin-like and transmembrane domains 3 | 2.92E-4 | 0.41 |
| ENSG00000101977 | MCF2 | MCF.2 cell line derived transforming sequence | 5.48E-3 | 0.14 |
| ENSG00000105419 | MEIS3 | Meis homeobox 3 | 1.84E-3 | 0.17 |
| ENSG00000135097 | MSI1 | musashi homolog 1 (Drosophila) | 7.68E-4 | 0.21 |
| ENSG00000205592 | MUC19 | mucin 19, oligomeric | 7.68E-4 | 0.03 |
| ENSG00000171428 | NAT1 | N-acetyltransferase 1 (arylamine N-acetyltransferase) | 0.02 | 0.87 |
| ENSG00000071051 | NCK2 | NCK adaptor protein 2 | 1.60E-9 | 2.94 |
| ENSG00000112333 | NR2E1 | nuclear receptor subfamily 2, group E, member 1 | 1.59E-3 | 0.16 |
| ENSG00000236409 | NRADDP | neurotrophin receptor associated death domain, pseudogene | 0.04 | 0.45 |
| ENSG00000166321 | NUDT13 | nudix (nucleoside diphosphate linked moiety X)-type motif 13 | 0.05 | 0.46 |
| ENSG00000257542 | OR7E5P | olfactory receptor, family 7, subfamily E, member 5 pseudogene | 1.04E-4 | 1.46 |
| ENSG00000089041 | P2RX7 | purinergic receptor P2X, ligand-gated ion channel, 7 | 1.57E-6 | 0.90 |
| ENSG00000160191 | PDE9A | phosphodiesterase 9A | 1.35E-5 | 0.27 |
| ENSG00000165495 | PKNOX2 | PBX/knotted 1 homeobox 2 | 0.02 | 0.08 |
| ENSG00000141744 | PNMT | phenylethanolamine N-methyltransferase | 0.02 | 0.26 |
| ENSG00000165807 | PPP1R36 | protein phosphatase 1, regulatory subunit 36 | 1.19E-5 | 0.37 |
| ENSG00000067606 | PRKCZ | protein kinase C, zeta | 6.94E-3 | 0.30 |
| ENSG00000138669 | PRKG2 | protein kinase, cGMP-dependent, type II | 1.03E-14 | 0.75 |
| ENSG00000119608 | PROX2 | prospero homeobox 2 | 0.04 | 0.19 |
| ENSG00000148300 | REXO4 | REX4, RNA exonuclease 4 homolog (S. cerevisiae) | 2.93E-4 | 1.14 |
| ENSG00000232399 | RP11-1286E23.8 | ubiquitin carboxyl-terminal hydrolase 17-like | 3.27E-3 | 0.30 |
| ENSG00000228470 | RP11-176D17.3 | Novel processed transcript | 0.02 | 1.88 |
| ENSG00000224616 | RP11-305E17.6 | Novel antisense | 5.43E-3 | 1.41 |
| ENSG00000253549 | RP11-317J10.2 | Novel antisense | 1.59E-3 | 0.48 |
| ENSG00000260281 | RP11-329J18.2 | Novel antisense | 0.01 | 0.65 |
| ENSG00000263041 | RP11-355F22.1 | Known processed transcript | 2.94E-5 | 1.49 |
| ENSG00000258743 | RP11-406A9.2 | Novel lincRNA | 0.04 | 0.18 |
| ENSG00000229109 | RP11-439K3.1 | Putative antisense | 8.69E-5 | 1.91 |
| ENSG00000251584 | RP11-440I14.3 | Putative lincRNA | 0.03 | 0.44 |
| ENSG00000262370 | RP11-473M20.9 | Novel lincRNA | 0.05 | 0.31 |
| ENSG00000255773 | RP11-566K11.1 | Novel lincRNA | 0.03 | 0.14 |
| ENSG00000255050 | RP11-661A12.9 | Novel antisense | 0.04 | 0.27 |
| ENSG00000249955 | RP11-6E9.4 | Novel antisense | 1.19E-3 | 1.82 |
| ENSG00000253479 | RP11-744J10.3 | Novel lincRNA | 0.05 | 0.40 |
| ENSG00000255197 | RP11-750H9.5 | Novel antisense | 0.04 | 0.28 |
| ENSG00000257052 | RP11-881M11.2 | Novel antisense | 0.03 | 0.43 |
| ENSG00000230990 | RP4-734C18.1 | Putative lincRNA | 0.04 | 0.22 |
| ENSG00000236908 | RP5-1063M23.2 | Novel lincRNA | 0.02 | 0.13 |
| ENSG00000228265 | RP5-1125A11.1 | Putative lincRNA | 0.03 | 0.29 |
| ENSG00000260548 | RP6-24A23.6 | Putative protein coding | 3.27E-3 | 0.95 |
| ENSG00000198794 | SCAMP5 | secretory carrier membrane protein 5 | 0.04 | 0.10 |
| ENSG00000185437 | SH3BGR | SH3 domain binding glutamic acid-rich protein | 2.05E-3 | 0.95 |
| ENSG00000158296 | SLC13A3 | solute carrier family 13 (sodium-dependent dicarboxylate transporter), member 3 | 0.04 | 0.10 |
| ENSG00000110628 | SLC22A18 | solute carrier family 22, member 18 | 1.68E-3 | 0.18 |
| ENSG00000147606 | SLC26A7 | solute carrier family 26, member 7 | 2.54E-3 | 0.21 |
| ENSG00000101222 | SPEF1 | sperm flagellar 1 | 0.04 | 0.16 |
| ENSG00000165730 | STOX1 | storkhead box 1 | 1.14E-3 | 2.26 |
| ENSG00000166069 | TMCO5A | transmembrane and coiled-coil domains 5A | 6.83E-6 | 0.20 |
| ENSG00000198133 | TMEM229B | transmembrane protein 229B | 6.05E-5 | 0.14 |
| ENSG00000178297 | TMPRSS9 | transmembrane protease, serine 9 | 6.79E-3 | 0.14 |
| ENSG00000159173 | TNNI1 | troponin I type 1 (skeletal, slow) | 2.94E-5 | 0.14 |
| ENSG00000176058 | TPRN | taperin | 0.04 | 0.36 |
| ENSG00000112041 | TULP1 | tubby like protein 1 | 0.01 | 0.21 |
| ENSG00000173610 | UGT2A1 | UDP glucuronosyltransferase 2 family, polypeptide A1, complex locus | 0.00 | 1.29 |
| ENSG00000171234 | UGT2B7 | UDP glucuronosyltransferase 2 family, polypeptide B7 | 0.02 | 0.17 |
| ENSG00000137766 | UNC13C | unc-13 homolog C (C. elegans) | 6.24E-14 | 0.39 |
| ENSG00000059145 | UNKL | unkempt homolog (Drosophila)-like | 1.46E-7 | 0.33 |
| ENSG00000075035 | WSCD2 | WSC domain containing 2 | 0.02 | 0.06 |
| ENSG00000220201 | ZGLP1 | zinc finger, GATA-like protein 1 | 0.03 | 0.14 |
| ENSG00000066185 | ZMYND12 | zinc finger, MYND-type containing 12 | 1.24E-3 | 0.60 |
| ENSG00000179909 | ZNF154 (19 58208734..58220579) | zinc finger protein 154 | 0.04 | 0.25 |
| ENSG00000138311 | ZNF365 | zinc finger protein 365 | 6.79E-3 | 0.05 |
| ^a^Genes with uniquely identified transcripts in cornea, q-value < 0.05, and higher RPKM values than SLCs. | | | | |
